# A review of the current state of natural biomaterials in wound healing applications

**DOI:** 10.3389/fbioe.2024.1309541

**Published:** 2024-03-27

**Authors:** Mojtaba Ansari, Ahmad Darvishi

**Affiliations:** Department of Biomedical Engineering, Meybod University, Meybod, Iran

**Keywords:** wound healing, wound dressing, skin tissue engineering, natural biomaterials, synthetic biomaterials

## Abstract

Skin, the largest biological organ, consists of three main parts: the epidermis, dermis, and subcutaneous tissue. Wounds are abnormal wounds in various forms, such as lacerations, burns, chronic wounds, diabetic wounds, acute wounds, and fractures. The wound healing process is dynamic, complex, and lengthy in four stages involving cells, macrophages, and growth factors. Wound dressing refers to a substance that covers the surface of a wound to prevent infection and secondary damage. Biomaterials applied in wound management have advanced significantly. Natural biomaterials are increasingly used due to their advantages including biomimicry of ECM, convenient accessibility, and involvement in native wound healing. However, there are still limitations such as low mechanical properties and expensive extraction methods. Therefore, their combination with synthetic biomaterials and/or adding bioactive agents has become an option for researchers in this field. In the present study, the stages of natural wound healing and the effect of biomaterials on its direction, type, and level will be investigated. Then, different types of polysaccharides and proteins were selected as desirable natural biomaterials, polymers as synthetic biomaterials with variable and suitable properties, and bioactive agents as effective additives. In the following, the structure of selected biomaterials, their extraction and production methods, their participation in wound healing, and quality control techniques of biomaterials-based wound dressings will be discussed.

## 1 Introduction

The skin is the largest and widest organ in the human body, making it more susceptible to disease, injury, and burns. An important disease is skin damage due to superficial and local wounds that require treatment (Nisar et al., 2023). Skin wounds are injuries in the form of tears, fractures, or abnormal defects that may be caused by pathological conditions, endogenous factors (e.g., diabetes, malignancy, vascular disease, etc.), physical trauma, and burns (Percival, 2002). Wound healing and repair are typically long-term, slow, complex, multifaceted, and dynamic biological processes. Simple and small wounds can heal on their own, but acute and large wounds heal slowly and heal, usually within 8–12 years, which may be affected by bacterial infections and dust particles during the wound healing process (Koehler et al., 2018; Shen et al., 2021). Therefore, the treatment of wounds against bacterial infections (e.g., infections caused by *Escherichia coli*, *Staphylococcus aureus*, and *Bacillus subtilis*) is one of the main problems in wound healing, which can hinder the healing process and threaten human health (Moritz et al., 2014; Neto et al., 2019). Wound dressing is a material that covers wounds and prevents secondary damage and infection. It also creates an environment for faster wound healing and prevents the build-up of bacteria and other infectious foreign substances in the barrier (Bankoti et al., 2017). In the past, coverings made from natural materials such as cotton were used to manage and repair wounds over time by absorbing fluids oozing from the wound and forming an outer layer called an eschar (Daristotle et al., 2020). However, this repair method delays the proliferation and migration of epidermal cells and consequently delays recovery. Over time, researchers have suggested using moist dressings to heal wounds and accelerate the healing process. Considering that moist wound dressings are applied to the wound for a long period of time, the materials used to accelerate the healing process must have physicochemical and biological properties suitable for the wound area. It covers the surface to be easily separated from the wound and releases antibiotics to treat microbial and bacterial infections (Singh et al., 2013; Costa et al., 2020; Dong and Guo, 2021). Various natural and synthetic polymers with different physical, chemical, and biological properties have been investigated and used to fabricate new wound dressings. Polymers derived from animals, plants, and microorganisms have become an ideal approach for wound healing due to their natural properties. Studies have shown that it is preferred for use as wound dressings over other materials due to its better compatibility (Gaspar-Pintiliescu et al., 2019; Bianchera et al., 2020). These biomaterials can be prepared in different ways in the form of hydrogel, film, nanofiber, foam, scaffold, etc. Hydrogel wound dressings based on natural biomaterials have been introduced as a new type of wound dressing due to their structural similarity to extracellular matrix (ECM). They create a moist, acidic environment to trigger the proliferation and growth of new cells, prevent tissue necrosis, relieve pain, and protect wounds (Dhivya et al., 2015; Asadi et al., 2021). Natural biomaterials such as silk, keratin, bacterial cellulose (BC), hyaluronic acid (HA), N-acetylglucosamine, gelatin (Gel), β-glucan, dextran (Dex), chitin/chitosan, and collagen (Col) are the most important and widely usage natural polymers used in wound dressings (Chinta et al., 2022).

## 2 Wound healing: history, requirements, process

### 2.1 History

Since ancient times, humans have realized that they need dressings to treat and heal wounds. For this reason, newer and more advanced wound dressings have been created over time. Before the 18th century, natural materials such as skin, flowers, and leaves of plants were used as dressings for wound healing, which caused harm such as wound inflammation by absorbing dust particles and bacteria (Yuan et al., 2023). Over time, scientists came to the conclusion that if a wound is scraped and disinfected in a dry environment, a structure called a scab will form under which wound healing occurs. Therefore, the issue of dry wound healing was studied (Wlaschin et al., 2019). However, researchers showed that dry wound healing has disadvantages such as the formation and accumulation of exudate on the wound and the need for frequent dressing changes, which lead to re-injury and prolonging the wound healing process (Broderick et al., 2020; Stupin et al., 2020; Chi et al., 2021; Xiao et al., 2021; Liu et al., 2022). For the first time in 1962, Dr. Winter proved in a series of animal experiments that compared to a dry environment, wound healing in a moist environment preserves body fluids in the wound, improves the ability of cell proliferation and migration, resolves the problem of wound scratching, and finally increases the wound healing rate. Therefore, the concept of moist wound healing was considered (Winter 1962). After that, Hinman et al. showed that moist wound healing can be studied and used in human experiments. Also, in addition to wound healing, it can play a role in healing pain, migration of new keratinocytes, and removal of damaged tissue and external factors from the wound. For this reason, scientists turned to the development of wound repairers based on the concept of wet wound repair (Hinrnan and Maibach, 1963; Nuutila and Eriksson, 2021).

### 2.2 Requirements

Various influential factors play a role in wound repair and healing, which are generally divided into two categories: systemic and intrinsic/local factors. The health and age of the patient are a subset of systematic factors. Intrinsic/local factors also include foreign bodies, infection, topical steroids, and decreased blood supply (Giannelli et al., 2014). Infection, as the main factor in wound healing, occurs when the surface layer of the skin (which protects the body as a natural barrier against foreign and pathogenic factors) is destroyed and the underlying tissue is exposed. The main characteristics of wound infection include increased swelling around the wound, causing and increasing pain and discomfort, increasing secretions and wound fluid, creating an unpleasant smell, and increasing the temperature of the wound site (Jerry et al., 2018; Mayor and Mills, 2018). Improper and insufficient care of wound infection slows down the healing and repair process of the wound and may also lead to the loss and amputation of the limb and even cause the death of the patient (Martín-Aspas et al., 2018). Therefore, it is necessary to consider factors such as the percentage and number of patients who suffer from various wounds every year and to estimate the costs of adequate care for wound healing (Carter et al., 2023). Studies in recent years have shown that in developing countries, about 1%–2% of the population suffers from chronic wounds every year. In the United States of America (United States), it is estimated that 4.5 million people are affected by these ulcers annually. A meta-analysis study showed that 2.2 out of every 1,000 people have chronic ulcers and 1.5 out of every 1,000 have chronic leg ulcers. Due to the increase of microorganisms and the spread of chronic non-communicable diseases, the costs of care and treatment are also affected by these factors and increase. Following these statements, during research conducted using Medicare health insurance in the United States, the annual cost for the care and treatment of these wounds has been estimated at 28 billion dollars (Nussbaum et al., 2018; Sen, 2021). Estimated costs for chronic wound care management in Europe are also enormous. According to research, about 4% of healthcare spending in the Scandinavian countries and 3% of the total budget of the United Kingdom National Health Service are allocated to managing chronic wounds (Olsson et al., 2019).

**FIGURE 1:**
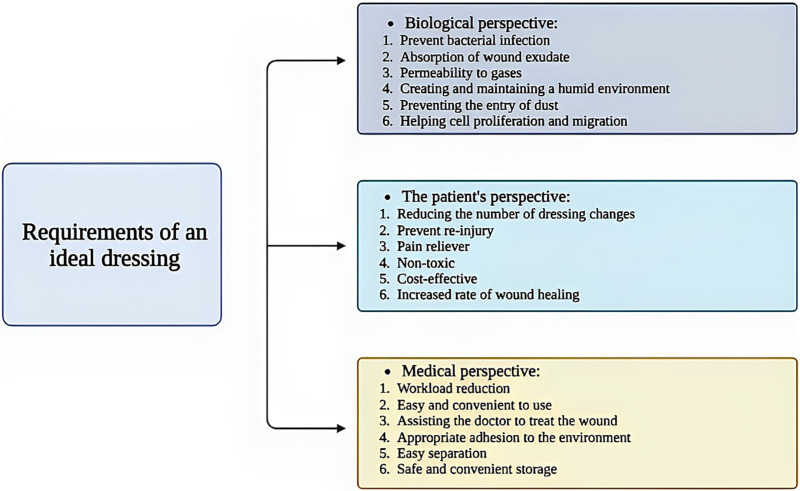
Schematic illustration of the requirements for a suitable wound dressing. Created with BioRender.com.

According to the contents of this section, to design a suitable wound dressing, various aspects and perspectives should be considered in order to receive the best feedback after treatment (Figure 1).

### 2.3 Wound healing process

**FIGURE 2:**
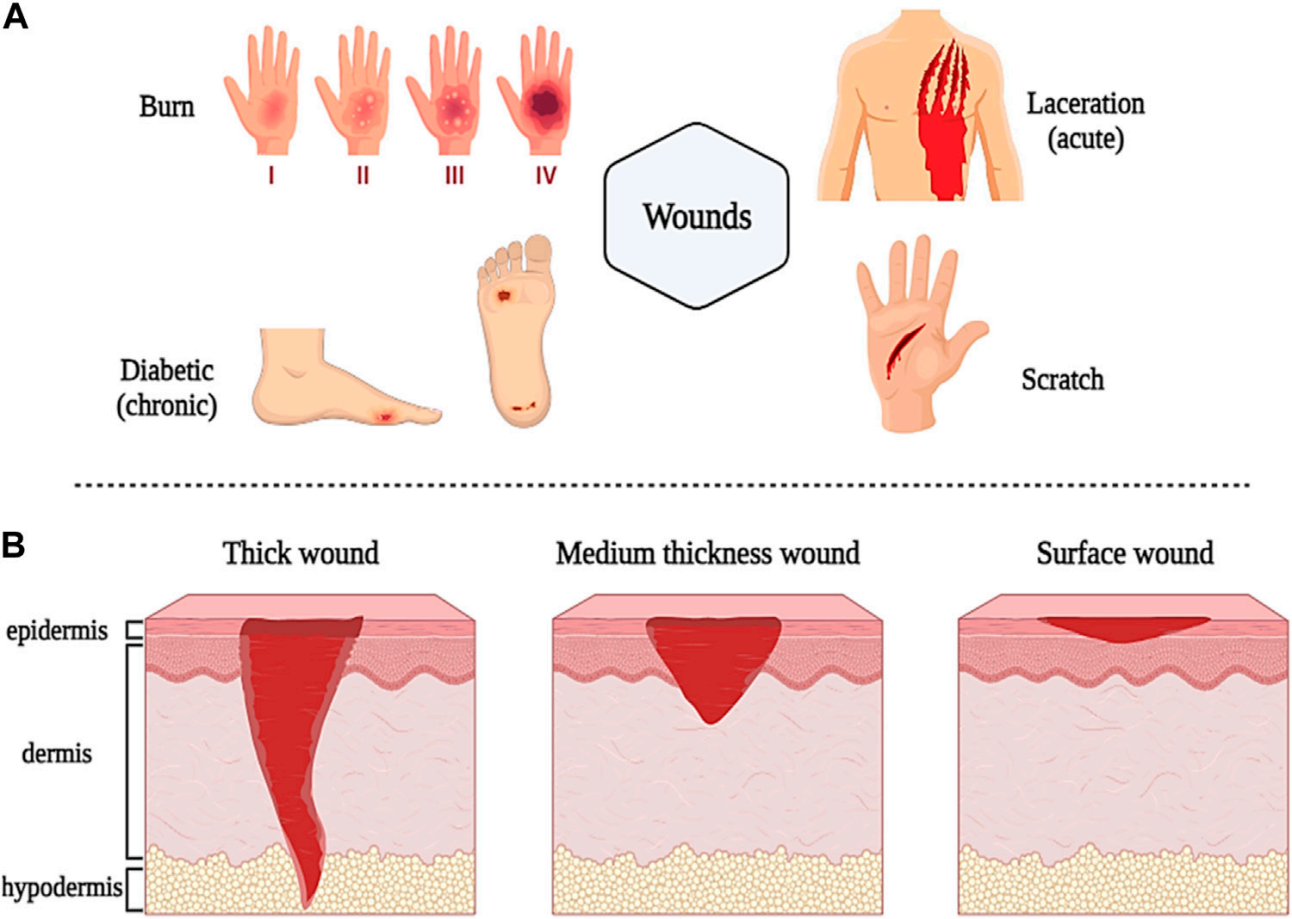
Schematic illustration of classification and different types of wounds based on sources **(A)** and depth **(B)**. Created with BioRender.com.

As mentioned in the introduction, a skin ulcer is a type of injury and disturbance in the physiological function and anatomical structure of the skin that occurs due to various factors such as disease, surgery, burns, and accidents. In general, wounds are classified according to the type and location of the injury, the depth of the wound, the loss or absence of tissue, and the duration of healing into all types of first-fourth degree burn wounds, lacerations, diabetic wounds, chronic wounds (slow healing time, more than 12 weeks), and acute wounds (rapid healing time, less than 12 weeks) (Figures 2A) (Schrementi et al., 2018; Patil et al., 2020). Thick wounds damage the epidermis, dermis, and underlying tissues (such as muscle and fat tissue), thinner wounds damage the epidermis and blood vessels, and small, superficial wounds damage only the epidermis (Figures 2B) (Singh et al., 2017a).

As one of the most common wounds in different ages, chronic wounds cover a large part of the world’s population, which is known as a “silent epidemic” (Graves et al., 2022). The difference between these wounds and other types is the duration of their healing and treatment. When a wound is not treated during the natural healing process and remains open for more than several months, it is classified as a chronic wound. Several factors contribute to the development of chronic ulcers, including poor nutrition and obesity, diabetes, lack of mobility, and aging (Raeder et al., 2020; Graves et al., 2022). Vulnerable people in long-term care facilities and home residents who are susceptible to injury are more exposed to chronic wounds. Since chronic wounds are open for a long time, other diseases also develop in patients, which complicates the recognition, management, and treatment of chronic wounds (Martinengo et al., 2019).

In the following, we will explain the natural wound healing process and introduce new strategies for healing different wounds.

**FIGURE 3:**
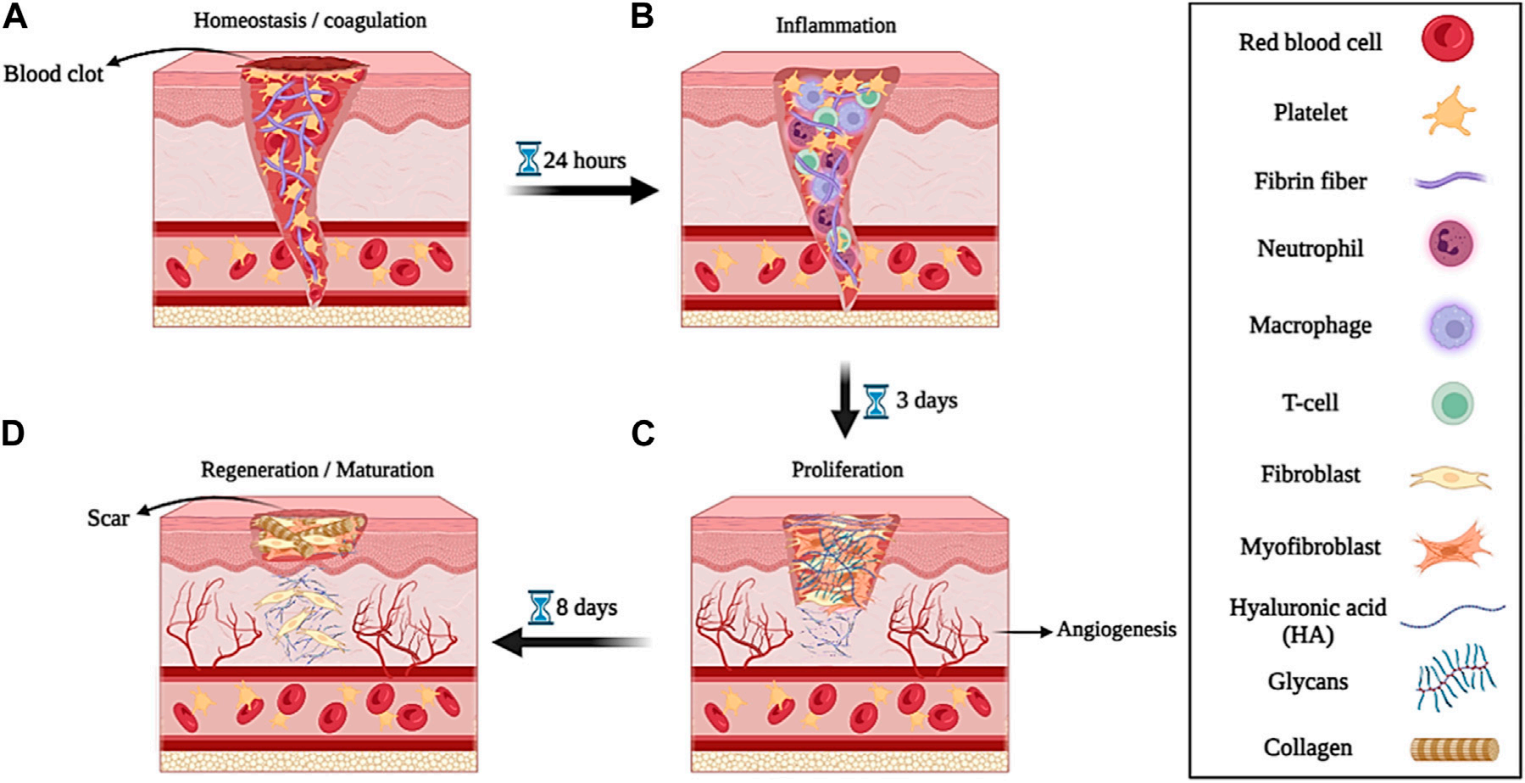
Schematic illustration of the wound healing process; **(A)** hemostasis or coagulation, **(B)** inflammation, **(C)** proliferation, and **(D)** regeneration or maturation. Created with BioRender.com.

When the skin is damaged due to various factors (such as disease, accident, etc.), the process of repairing and healing the wound begins. Biologically, the wound healing process is a regenerative, dynamic, and multi-step process that is mediated by various types of growth factors, cytokines, cells, various metal ions (such as Mg^2+^, Ca^2+^, Zn^2+^, etc.), and reactive molecular oxygen and nitrogen species (RONS) and it usually takes place in four phases or stages that lead to tissue growth and regeneration (Chin et al., 2019; Cho et al., 2019; Zhang et al., 2020a). The stages of wound healing that cause tissue regeneration include; **1)** homeostasis or coagulation, **2)** inflammation, **3)** proliferation, and **4)** regeneration or maturation (Figures 3A–D) (Stern and Cui, 2019).

➢ The first stage, called hemostasis or coagulation, occurs a few minutes after the injury when the body recognizes the damage to the blood vessels by forming a blood clot by accumulating platelets at the injured site, which prevents excessive bleeding (Figures 3A). The process of blood clot formation takes place when the components of the extracellular matrix (ECM) are in contact with each other (such as the interaction of collagen with platelets) and fibrinogen is converted into fibrin protein with the help of thrombin. Platelets, in the blood clotting process, produce and release several growth factors such as epidermal growth factor (EGF), platelet-derived growth factor (PDGF), basic fibroblast growth factor (bFGF), and transforming growth factor β (β-TGF), as well as cytokines such as tumor necrosis factor-α (α-TNF) and interleukin-1β (IL-1β) (Dargaville et al., 2013; Shoji et al., 2017). These growth factors and cytokines released after 6 h of injury attract neutrophils to start the initial matrix formation process for wound healing by lymphocytes, fibrins, and histiocytes. The process of primary matrix formation and wound healing takes place within 12 h after injury (Rousselle et al., 2019).
➢ In the stage of inflammation, which takes place to prevent microbial and bacterial infection, neutrophils move towards them to start the process of phagocytosis of microorganisms and bacteria in the damaged area (Figures 3B). Migration of neutrophils towards endothelial cells is carried out by formyl methionyl peptides, IL-1β, and α-TNF. This process, which occurs between 24 and 36 h after injury, is called the initial stage of inflammation (Negut et al., 2020). The stage of secondary inflammation begins after 48–72 h of injury. At this stage, monocytes and lymphocytes are differentiated into macrophages by cytokines and blood clotting factors and are transferred to the injury site. Macrophages use leukocytes and released cytokines to activate the inflammatory response in the wound environment. Also, macrophages induce cell apoptosis (such as neutrophils) and resolve inflammation. T lymphocytes are among the last cells that are attracted to the damaged area and they have shown their effect in wound healing by influencing and playing a role in collagen deposition and the development of capillary networks. Collagen deposition and capillary network development as well as fibroblast growth factors (FGF), PDGF, and vascular endothelial growth factor (VEGF) cause angiogenesis and the production of granulated tissues around the wound, which lead to wound contraction (Margolis et al., 2011; Wang et al., 2022).
➢ After the inflammatory phase, the proliferative phase of wound healing begins approximately 3 days after the injury and lasts for 2 weeks. This stage continues with the migration of fibroblasts to the wound site, angiogenesis, and epithelialization to change the fibronectin and fibrin network to form a new ECM. In this process, fibroblasts move to the injured site, where they produce hyaluronic acid (HA), fibronectin, glycans (such as proteoglycans and glycosaminoglycans), and collagen, which are all the main components required for the formation of ECM (Singh et al., 2010). Next, fibroblasts differentiate into myofibroblasts, which contain actin bundles that cause wound contraction by binding to collagen and fibronectin proteins in the ECM. The process of neovascularization also causes the growth and proliferation of blood vessels towards the damaged area. In the epithelialization process, epidermal keratinocytes play an essential role. They proliferate and differentiate in the epidermis and create a protective barrier against environmental and peripheral damage (Rousselle et al., 2019; Moltrasio et al., 2022). Therefore, the proliferation phase is divided into three phases: new ECM formation by fibroblasts, neovascularization, and epithelialization by keratinocytes (Figures 3C).
➢ The regeneration or maturation phase begins on the eighth day after the injury and continues for a long period of time (2 years in adults). This stage includes the repair and regeneration of a collagen network similar to healthy tissue and the formation and maturation of scar tissue (Figures 3D). However, the formed scar tissue is not capable of completely mimicking the properties of healthy tissue. For example, the collagen structure of scar tissue is not the same as the collagen structure of healthy skin. Also, mature scar tissue mimics only 80% of the tensile strength of healthy skin tissue (Scuderi et al., 2008; Sinno and Prakash, 2013).

## 3 Biomaterials used in wound healing

Different natural and synthetic biomaterials with diverse properties are used to make new wound repair materials. In this section, we will review some common natural/synthetic biomaterials-based wound dressings for skin tissue engineering (Table 1).

**TABLE 1:** Summary of studies conducted in the field of biomaterials-based wound dressings.

Biomaterials	Study type	Targeted application	Major outcomes	References.
Silk fibroin (SF) films	*In vitro*	Trauma	SF-based films promoted the growth and secretion of VEGF from endothelial cells and had no adverse effect on it	Liu et al. (2010)
*Bombyx mori* silkworm-derived SF hydrogels	*In vivo*	Full-thickness skin excision	SF hydrogels showed a sustained release of FGF1 and induced the proliferation and migration of L929 fibroblast cells in mice with skin damage and treated them	He et al. (2019a)
methacrylated SF (SFMA), methacrylated chitosan (CSMA), tannic acid (TA)	*In vivo*	Full-thickness skin defect	The presence of TA increases the mechanical performance of the hydrogel by five times. High ability of the hydrogel in healing and healing the wounds of mice	He et al. (2020)
Fibrin-chitin embedded gelatin nanoparticle- hydrogel	*In vivo*	Cardiac surgery wounds	The prepared nanocomposites reduce bleeding in the shortest time and cause blood clots	Sundaram et al. (2018)
chitosan (CS)/PVA/fibrin, sodium alginate (SA), gelatin-three layered hydrogels	*In vitro*	Burn wounds	Excellent antibacterial activity, high water absorbant	Talukder et al. (2021)
Fibrin-agarose engineered skin substitutes	*In vivo*	Skin tissue engineering	adequate biomechanical properties and appropriate biocompatibility, engineered skins showed better cell differentiation and skin structure regeneration after 30 days of implantation in the *in vivo* environment compared to samples implanted for 10 or 20 days	Carriel et al. (2012)
grafted Fibrin-agarose skin substitute (UGRSKIN)	Clinical trial	Severe burn wounds	Well-differentiated keratinocytes, rapid and high differentiation of the epidermis, increased elastic fibers, collagens and proteoglycans (such as decorin) as the group control on days 60–90, and the formation of blood vessels by CD31 and the expression of SMA in the grafted skin were higher than in the control group, while the formation of lymphatic vessels was more abundant on the 90th day	Martin‐Piedra et al. (2023)
Keratin films coated with cystine particles	*In vitro*	Wound healing	Suitable hydrophobic properties, high strength in wet environments and favorable flexibility	Mi et al. (2019)
Keratin/polyvinylpyrrolidone (PVP)-based hydrogels incorporated with lavender extract	*In vitro*	Wound healing	High mechanical strength and swelling ratio, favorable antibacterial properties	Tajik et al. (2021)
Poly (γ-glutamic acid)-keratin hydrogels	*In vitro*	Wound healing and cartilage repair	This hydrogel gives an elastic modulus of 4.5 kPa and an approximate swelling rate of 2500%	Bajestani et al. (2020)
Oxidized bacterial cellulose (OBC), chitosan (CS), and collagen (COL) nanocomposites	*In vivo*	Rapid hemostasis and wound healing	High antibacterial properties, blood clotting capability, and increasing the hemostatic effect and wound healing	Yuan et al. (2019)
BC, TEMPO, Ag NPs	*In vitro*	Wound healing	Excellent antibacterial activity against E.coli and S.aureus (100% and 99.2% respectively), and high biocompatibility (cell viability >95%)	Wu et al. (2018a)
HA-based films grafted with pullulan (Pu)	*In vivo*	Skin regeneration and wound healing	High swelling ratio, the binding of Pu to HA increases the stability of HA-based films, porosity between 29.36 ± 73.13 μm, which is suitable and compatible for cell migration, and excellent biocompatibility	Li et al. (2018)
HA-EDA hydrogels	*In vitro*	Angiogenesis and wound healing	After 5 days of incubation, 50% of VEGF was retained in the hydrogel, which stimulated the proliferation of human vascular endothelial cells (HUVEC) (which is effective in the angiogenesis process during wound healing)	Fiorica et al. (2018)
N-acetylglucosamine (GlcNAc)	*In vivo*	Angiogenesis and wound healing	Collagen synthesis and fibroblast proliferation increased and improved the wound angiogenesis process. The rate of wound closure was favorably increased	Ashkani-Esfahani et al. (2012)
GlcNAc, and CS filament	*In vitro*	Absorbable surgical suture	The mechanical resistance decreased after adding GlcNAc to CS, low cytotoxicity, and caused pain relief, reduced surgical infection, and finally improved the surgical wound healing process	da Silva et al. (2019)
collagen/glycosaminoglycan (COL/GAG)-based cellular scaffold	*In vitro*	Wound healing and skin tissue regeneration	The produced scaffolds increase the adhesion and proliferation of AFS cells, which is recognized as an attractive cell source in wound healing and skin tissue regeneration	Ansari et al. (2018a)
Titatnium dioxide nanoparticles/Gelatin composite	*In vivo*	Wound healing	The produced composite promotes the survival and adhesion of fibroblast cells between 72 h and 7 days. Additionally, it was increased angiogenesis, tissue granulation, and re-epithelialization without causing toxicity	Nikpasand and Parvizi (2019)
Dex, HA, and β-cyclodextrin (β-CD)-based hydrogel	*In vivo*	Burn wounds	The produced hydrogel increased biocompatibility, reduced wound inflammation, increased microvascular growth, and ultimately burn wound healing	Wang et al. (2019a)
chitosan/guar gum/peppermint essential oil (CS/GG/PEO) antibacterial hydrogel	*In vivo*	Burn wounds	CS/GG/PEO hydrogel significantly improves the angiogenesis process, collagen fiber thickness, re-epithelialization, and wound contraction (90% on day 22)	Ansari et al. (2023)
yeast β-glucan ointment	Clinical trial (33 patients)	Second and third-degree burn wounds	Patients who received β-glucan ointment showed better wound healing (RR = 4.34; 95% Cl; 0.73 to 25.67; *p* = 0.11)	Abedini et al. (2022)
Polycaprolactone (PCL) fibers, and casein	*In vivo*	Diabetic wounds	SEM images showed fibers with an average diameter of 1.4 ± 0.5 μm (suitable for covering wounds), casein-loaded PCL fibers showed more than 90% viability of mouse fibroblast cells. Casein-loaded fibers showed follicle regrowth and increased granulation tissue growth in histological tests. Casein-containing fibers express lower levels of IL-6, TNF-α, TGF-β, IL-1β, and NF-kB, which results in more beneficial healing	Ahmed et al. (2023)

### 3.1 Natural biomaterials in wound healing

Natural biomaterials, due to their diverse and favorable properties such as appropriate biodegradability, excellent biocompatibility, easy manufacturing process, good adhesion, high absorption and permeability, and finally high capacity for regeneration and repair of damaged tissue, have received a lot of attention in skin tissue engineering and wound healing (Kamoun et al., 2017; Chinta et al., 2022). Additionally, the native nature of these biomaterials as well as the ability to extract them from the biomass produced in food, textile, and other industries have made them more accessible (Isaza-Perez et al., 2020). Thus, natural biomaterials or their extraction from biomasses can be preferred over synthetic ones for environmental reasons. In the following, we will discuss some of the natural biomaterials used to make wound repair materials such as silk, keratin, bacterial cellulose, and hyaluronic acid.

#### 3.1.1 Silk-based wound dressings

**FIGURE 4:**
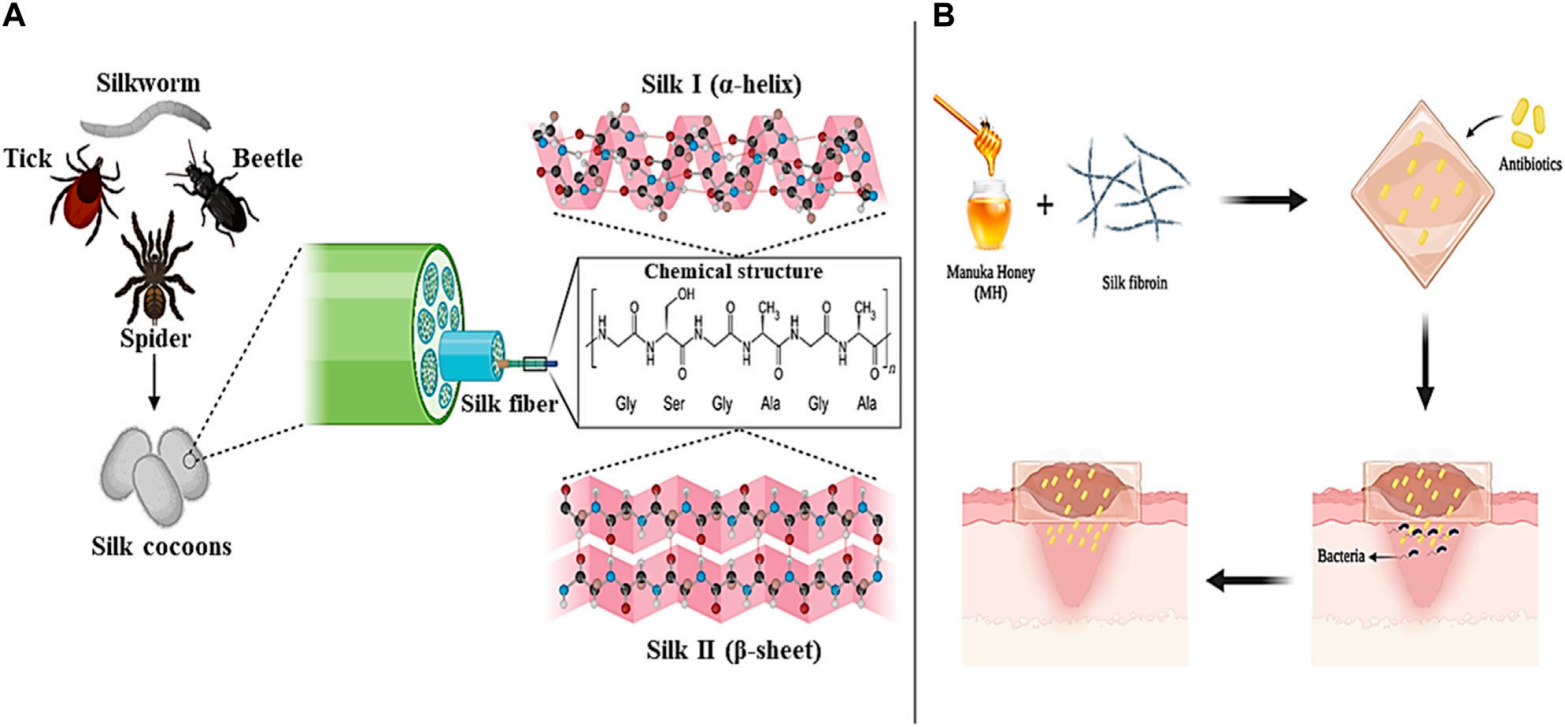
Silk panel; **(A)** sources and chemical structure, **(B)** Silk/MH-based antibiotic loaded wound dressing. Created with BioRender.com.

Silk is a type of protein with a fibrous structure that is obtained from various natural sources such as spiders, silkworms, beetles, and ticks (Sutherland et al., 2010). Silk fibroin (SF), mainly secreted from a domesticated silkworm called *Bombyx mori*, consist of a repeating chain structure with a light chain weighing 26 kDa and a heavy chain weighing 390 kDa (1:1 ratio) with a diameter of the fibers between 10 and 25 mm. The heavy chain of Silk fibroin includes hydrophobic sequences of glycine and alanine (Gly-Ala-Gly-Ala-Gly-Ser) and a non-repetitive and short hydrophilic part, which is why it exhibits amphiphilic properties (Figures 4A) (Asakura et al., 2020).

Silk can be produced in different formats such as electrospun fibers, particles, hydrogels, and films. The silk must first be extracted from the cocoon to produce these forms. During the regeneration process (silk extraction), an aqueous solution is obtained from which the amorphous structure of silk (silk I) with α-helix content and the crystalline structure of silk (silk II) with β-sheet content are prepared (Perotto et al., 2017). Silk I is the short chain, amorphous and hydrophilic, and silk II is the long chain, heavy, crystalline, and hydrophobic. To convert silk from amorphous to crystalline, two methods of water vapor treatment and alcohol treatment are usually used. Compared to alcohol treatment, crystallization with the help of water vapor is a more controlled, slower, and calmer method that can obtain different crystallinity degrees of silk by changing the treatment time (Pignatelli et al., 2018).

Silk is also effective in the molecular mechanism and cell signaling pathways in the wound healing process. For example, silk can control the expression levels of anti-inflammatory cytokines IL-10 and pro-inflammatory cytokines IL-1a and IL-6 (overexpression of each of which impairs the wound healing process) (Ju et al., 2016). Also, SF-based wound repair agents can increase the speed of wound healing by controlling the expression of cyclin D1, NIH3T3 cell fibronectin, VEGF, vimentin and stimulating the NF-kB signaling pathway (which controls cell attachment and growth) (Park et al., 2018). In addition, SF stimulates cell migration through activation of various signaling pathways such as PI3K, MEK, and JNK kinases and phosphorylation of JNK1/2 and ERK1/2 kinases. The activation of these signaling pathways increases the phosphorylation of c-jun protein, which is one of the key factors in wound healing (Martinez-Mora et al., 2012). For example, in one study it was shown that SF-based films promoted the growth and secretion of VEGF from endothelial cells and had no adverse effect on it. Furthermore, adverse effects of SF film on the production and secretion of growth factors VEGF, PDGF, angiopoietin 1 (Ang-1), and fibroblast growth factor 2 (FGF-2) were not observed (Liu et al., 2010). In another study, hydrogels based on *Bombyx mori* silkworm-derived SFs loaded with human acidic fibroblast growth factor 1 (FGF1) were designed, and *in vitro* and *in vivo* studies were conducted on them. The results showed that SF hydrogels showed a sustained release of FGF1 and induced the proliferation and migration of L929 fibroblast cells in mice with skin damage and treated them (He et al., 2019a).

One of the important factors that should be considered in the management of wound healing is preventing the increase of bacterial and microbial infections and delaying the wound healing process (Unnithan et al., 2012). In addition to the favorable properties of biocompatibility, biodegradability, and hydrophilicity, SFs have the ability to create antibacterial and antimicrobial effects (alone or in combination with other biomaterials) in the wound environment. For example, in a study, SF electrospun nanofibers were combined with the natural substance Manuka honey (MH) and a type of antibiotic suitable for wound treatment was loaded into them. The evaluation results showed that the combination of SF nanofibers with MH led to an increase in the antimicrobial and antibacterial effects of the produced dressing and improved wound healing without side effects. Also, MH in the dressing composition improved skin tissue regeneration by regulating the release of cytokines and inducing an immune response against infection (Figures 4B) (Yaghoobi et al., 2013; Yang et al., 2017). In another study, He et al. (2020) produced a new hydrogel based on methacrylated SF (SFMA) together with methacrylated chitosan (CSMA) and tannic acid (TA) through a two-step process of photopolymerization and incubation in TA solution for effective wound healing. Examination of the mechanical properties showed that the presence of TA increases the mechanical performance of the hydrogel by five times. Also, the effect of SFMA/CSMA/TA hydrogel on the healing of thick wounds infected with *Staphylococcus aureus* bacteria that were created in the body of a mouse model was investigated and the results proved and confirmed the high ability of the hydrogel in healing and healing the wounds of mice. According to the studies conducted and the results obtained, SFs can be introduced as suitable and promising options in the production of biocompatible and biodegradable wound dressings with antimicrobial and anti-inflammatory effects in modern wound healing applications.

#### 3.1.2 Fibrin-based wound dressings

Fibrin is the main protein involved in blood clotting, which is derived from fibrinogen (an inactive glycoprotein in the blood flow) (Bayer, 2022). Fibrinogen is converted to fibrin monomers by thrombin. In this process, thrombin enzymatically cleaves fibrinopeptides A and B (FpA and FpB respectively) and produces fibrin monomers. Fibrin polymerization consists of three stages. In the first step, a double-stranded trimer is created from fibrin monomers. In the next step, longitudinal oligomers of fibrin double-stranded trimers grow until their length reaches 600–800 nm. In this case, they are called protofibrils. Finally, protofibrils begin to assemble laterally, which leads to the formation of a native, insoluble, viscoelastic, and biocompatible fibrin hydrogel (Bayer, 2022; Sanz-Horta et al., 2023).

Today, the great effect of fibrin in wound healing is well known. This natural biomaterial plays an essential role in homeostasis, inflammation, skin care against infections, and tissue regeneration. Therefore, wound dressings based on fibrin or fibrin/other biomaterials are classified as one of the most effective (Downer et al., 2023). The unique properties of fibrin make it possible to produce customized and innovative dressings in different shapes and designs with special structures and attributes (Su et al., 2023). In one study, researchers designed a fibrin-chitin hydrogel system and embedded gelatin nanoparticles loaded with tigecycline. *In vivo* studies on the damaged liver and femoral artery of a mouse model showed that the prepared nanocomposites reduce bleeding in the shortest time and cause blood clots that can be used in cardiac surgery applications (Sundaram et al., 2018). In another study, three-layer electrospun hybrid dressings were designed and evaluated for the treatment of burn wounds. In this dressing, the underlying layer was a combination of chitosan (CS)/PVA/fibrin designed to resist and prevent bleeding. The middle layer was composed of PVA/sodium alginate (SA) compounds, which gave antibacterial properties to the dressing. The upper layer was made of gelatin due to its excellent hydrophilicity. The results of the assays showed that this three-layer hybrid dressing is promising for burn wound application (Talukder et al., 2021). Carriel et al. Using fibrin-agarose biomaterials and cells obtained from a human skin biopsy, engineered skin substitutes were produced and evaluated in *ex vivo* and *in vivo* environments. Engineered skins were transplanted into immunodeficient mice and were tested and analyzed on days 10, 20, 30, and 40, respectively. The results confirmed the adequate biomechanical properties and appropriate biocompatibility of fibrin-agarose-engineered skins. Engineered skins showed better cell differentiation and skin structure regeneration after 30 days of implantation in the *in vivo* environment compared to samples implanted for 10 or 20 days. Finally, the researchers confirmed that the fibrin-agarose-engineered skins can regenerate the histological structure of native human skin after long-term implantation *in vivo* (Carriel et al., 2012). Martin‐Piedra et al. investigated the effects of a grafted fibrin-agarose skin substitute model (UGRSKIN) on severe burn patients for 3 months. The results of the investigations showed that the grafted model was similar to the native human skin from the 30th day onwards. Moreover, the grafted model showed the formation of layers of well-differentiated keratinocytes that show proper expression of plakoglobins, involucrins, claudins, filaggrins, and CK5, CK8, CK10, similar to the control group, which confirmed the rapid and high differentiation of the epidermis. In addition, the grafted skin model showed an increase in elastic fibers, collagens and proteoglycans (such as decorin) as in the control group on days 60–90. The expression of blood vessels by CD31 and the expression of SMA in the grafted skin were higher than in the control group, while the formation of lymphatic vessels was more abundant on the 90th day (Martin‐Piedra et al., 2023).

#### 3.1.3 Keratin-based wound dressings

**FIGURE 5:**
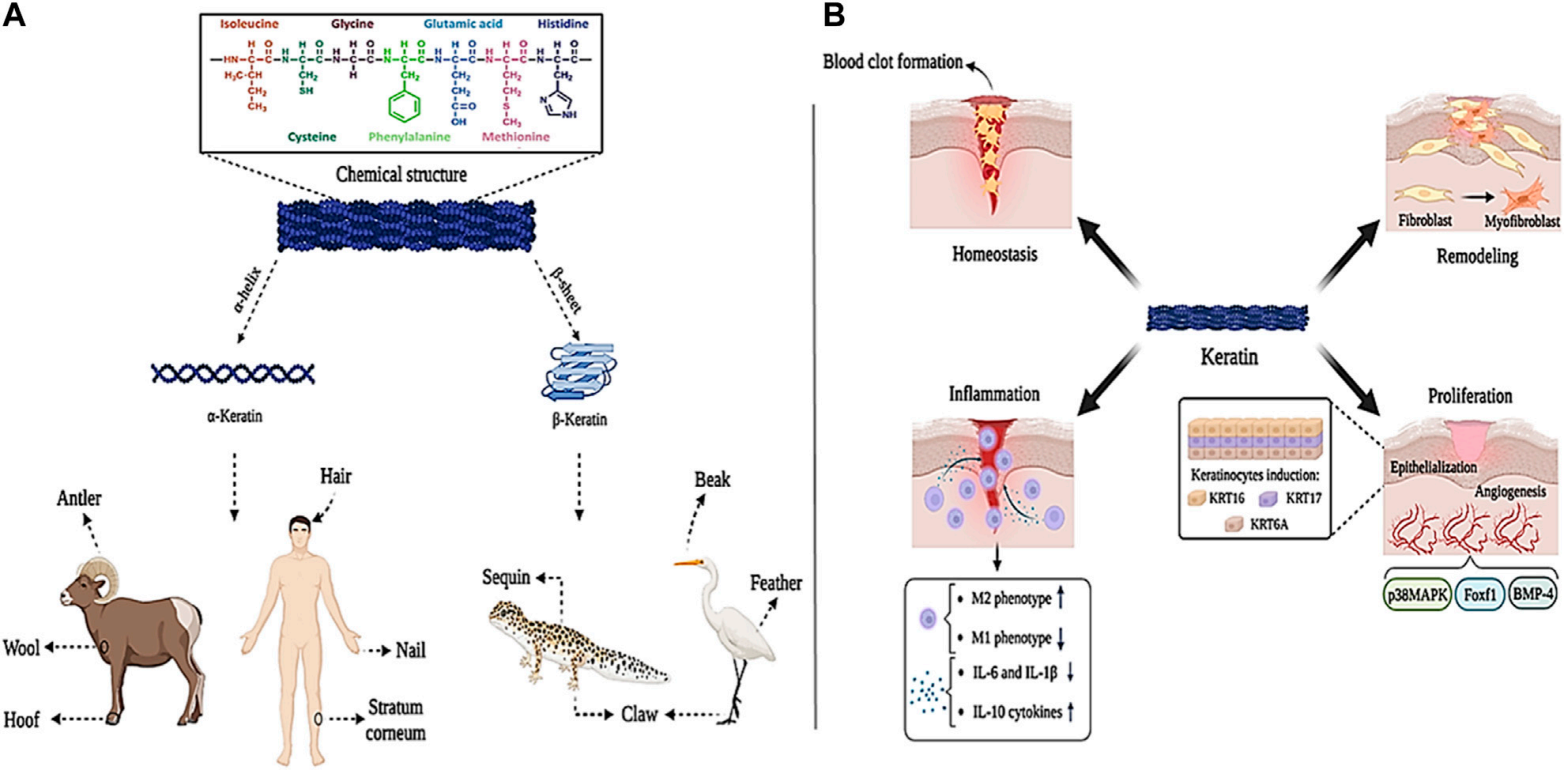
Keratin panel; **(A)** sources and chemical structure, **(B)** its role in wound healing process. Created with BioRender.com.

The word keratin is derived from the Greek word “Kera” meaning “horn”. Keratin is a structural protein derived from different parts of the mammalian body such as nails, hair, feathers, horns, wool, and epithelium. Structural observations have shown that keratin contains a large amount of amino acids like glycine, proline, cystine, alanine, and serine, while a small amount of histidine, tryptophan, lysine, and methionine amino acids are found in it (Korniłłowicz-Kowalska and Bohacz, 2011). Polypeptide chains in the sequence of amino acids are placed in different forms based on charge, size, and polarity, which leads to the formation of α-helix and β-sheet structures in keratin, which are called α-keratin and β-keratin, respectively (Lazarus et al., 2021). α-keratin, with a molecular mass between 40 and 68 kDa, is mostly found in hair, nails, wool, antlers, hoofs, and stratum corneum of mammals. Meanwhile, β-keratin sheets with a molecular mass of about 10–22 kDa are much smaller than α-keratin and are found in the hard tissues of birds such as beaks, claws, and feathers, as well as in the claws of reptiles and their sequins (Figures 5A) (Wang et al., 2016). Because of their different structures, *α* and *β* keratin exhibit different mechanical properties, as shown, Young’s modulus of α-keratin fibers is 2 GPa and their breaking strain is approximately 45%. This is while β-keratin with higher modulus and tensile strength (3.6 GPa and 203 MPa, respectively), is denser and shows more strength (Donato and Mija, 2019).

Annually, millions of tons of keratin-containing biomass are produced by the textile and food industries. Therefore, new efficient techniques for extracting creatine from these biomasses are an indispensable solution to accessing this biomaterial (Vineis et al., 2019). Keratin extraction is possible through the cleavage of interchain hydrogen bonds and disulfide covalent bonds. Generally, two methods of hydrolysis and denaturation are used to extract creatine. In the hydrolysis method, a mixture of polypeptides, low molecular weight proteins, and low sulfur content is prepared in powder form, which is used as a filler for other biopolymers. Denaturing method produces keratin with amino acid composition and native molecular weight distribution, which can be prepared in different forms such as sponges, films, and nanofibers (Aluigi et al., 2014; Bhavsar et al., 2017).

Studies have shown that keratin proteins are effective in wound healing. Keratin is effective in the hemostasis phase through diffusion to the wound environment during blood clot formation and reducing plasma clotting time. Also, keratin regulates M2 macrophage (which is anti-inflammatory and supports tissue regeneration), decreases the production of M1 macrophage (which leads to tissue destruction), decreases the production of pro-inflammatory cytokines IL-6 and IL-1β, and increases the production of anti-inflammatory cytokine IL-10, which effectively speeds up tissue regeneration and wound healing (Rahmany et al., 2013; Fearing and Van Dyke, 2014; Waters et al., 2018). In addition, keratin causes wound closure and epithelialization by inducing KRT16, KRT17, and KRT6A keratinocytes. Moreover, keratin through BMP-4, Foxf1, and p38MAPK signaling pathways is effective in the process of angiogenesis, hematopoiesis, and adhesion between mesoderm and endoderm layers (Vijayaraj et al., 2010; Kim et al., 2019; Botelho et al., 2021). Keratin is also involved in the formation of myofibroblasts (which are involved in the production of the primary ECM) by upregulating the expression of type IV and VII collagen (Figures 5B) (Gonzalez et al., 2016).

Keratin-based wound repair agents are being fabricated and evaluated in various forms such as films, cross-linked hydrogels, and electrospun nanofibers (Kossyvaki et al., 2020; Suarato et al., 2020; Sanchez Ramirez et al., 2021a; Sanchez Ramirez et al., 2021b). For example, in one study of biological and physicochemical properties of pure keratin and keratin/polysaccharide composite polymer films coated with nonwoven dressing materials such as keratin/sodium alginate (CFK/SA/NW), chicken feather keratin (CFK/NW) and keratin/chitosan (CFK/CS/NW) were compared with each other. The results of *in vitro* analysis showed that the addition of different polysaccharides increased the antibacterial effects of the produced films by inhibiting gram-negative and gram-positive bacteria (inhibition zone 2 cm). Furthermore, animal evaluations showed that CFK/CS/NW, CFK/SA/NW, and CFK/NW films reduced wound healing time by 35, 26, and 9%, respectively (Shanmugasundaram et al., 2018). In another study, the wet stability of keratin films coated with cystine particles was investigated for wound healing applications. The results showed that the produced films can be used in wound healing due to their suitable hydrophobic properties, high strength in wet environments and favorable flexibility (Mi et al., 2019). In one study by Tajik et al., keratin/polyvinylpyrrolidone (PVP)-based hydrogels were cross-linked by UV light irradiation. Then, the lavender extract was loaded into the keratin/PVP hydrogel. The results of the evaluations showed that the hydrogel with a higher ratio of PVP to keratin (ratio 1:3) exhibits greater mechanical strength and swelling ratio. Also, the presence of lavender extract increased the antibacterial properties of the hydrogel, which improved its application in wound healing (Tajik et al., 2021). In another study, a keratin/γ-polyglycolic acid (γ-PGA)-based hydrogel was fabricated by a chemical reaction between carboxyl and amino groups. Examination of mechanical properties and swelling ratio showed that this hydrogel gives an elastic modulus of 4.5 kPa and an approximate swelling rate of 2500%. This high strength and extraordinary swelling rate, together with other properties of keratin, such as softness and transparency, led researchers to use keratin-based hydrogels for cartilage tissue repair applications (Bajestani et al., 2020).

According to the results, due to its favorable antibacterial properties, biocompatibility, hydrophilicity, suitable mechanical strength, and high flexibility in manufacturing methods, keratin can be considered a suitable candidate for fabricating wound dressings.

#### 3.1.4 Bacterial cellulose-based wound dressings

**FIGURE 6:**
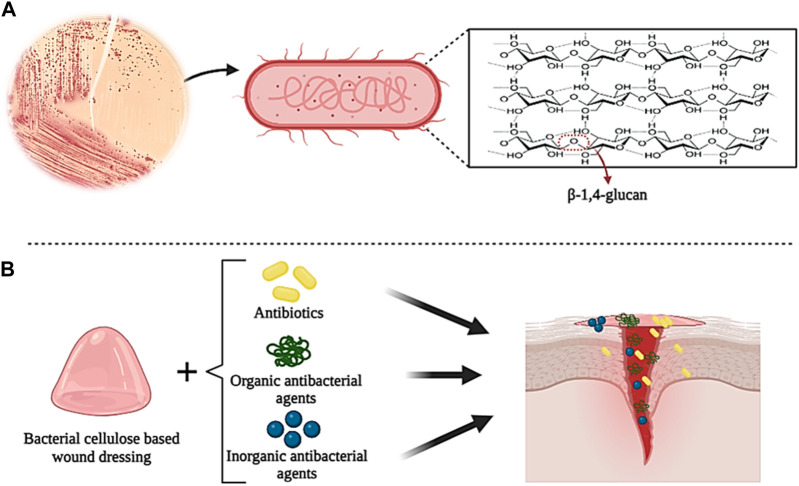
Schematic illustration of the chemical structure of bacterial cellulose **(A)** and its application in wound healing **(B)**. Created with BioRender.com.

Bacterial cellulose (BC) is a natural polymer that was first produced in 1886 by Brown. BC is formed by different bacterial strains with a 3D network structure connected through linear β-1,4-glucan cellulose chains (Figures 6A) (Sulaeva et al., 2015; Ahmed et al., 2020). This process is carried out by various bacterial species such as Agrobacterium, Stobacter, Sarcina, Rhizobium, etc., which can produce cellulose in a biosynthesis pathway that includes the secretion of various polysaccharides from carbon sources (such as glucose, maltitol, lactose, fructose, sucralose, etc.). However, BC can be prepared by a cell-free system in which the produced BC retains its cell extract but lacks the complete cell structure (Cheng et al., 2017; Dórame-Miranda et al., 2019; Fernandes et al., 2020; Ul-Islam et al., 2020). The unique properties of BC, such as proper flexibility, high tensile strength, permeability to liquids and gases, high water absorption and retention capacity, excellent biocompatibility in the biological environment, blood compatibility, non-cytotoxicity, etc., make it a desirable biomaterial in tissue repairers (Wu et al., 2018b; Inal and Mülazımoğlu, 2019; Graça et al., 2020). However, BC does not have antibacterial activity, which limits its use as wound dressings (Nuutila and Eriksson, 2021). To overcome this problem, many studies have proposed combining BC with biomaterials with antibacterial properties to produce antibacterial wound dressings. BC, due to its high surface area and porosity, can release drugs (such as antibiotics), organic and inorganic antibacterial agents, and other biological substances (Figures 6B) (Buruaga-Ramiro et al., 2020).

In studies on the preparation of BC-based composite wound dressings with antibacterial properties, three main methods have been used: combination with organic antibacterial agents, combination with inorganic antibacterial agents, and addition of antibiotics (Orlando et al., 2020; Zheng et al., 2020; Raut et al., 2023). Among organic antibacterial materials, natural polymers such as chitosan, collagen, and curcumin, have been used more due to their excellent biodegradability and biocompatibility (Zheng et al., 2020). For example, in one study, polymer blends based on BC modified by low molecular weight chitosan (Chi) were prepared for the controlled release of ciprofloxacin (CIP). The results showed that the presence of Chi increased the antibacterial activity of CIP in the polymer mixture (Cacicedo et al., 2020). Also, Yuan et al. (2019) prepared a novel hemostatic nanocomposite based on oxidized bacterial cellulose (OBC), chitosan (CS), and collagen (COL) (OBC/CS/COL). The research results showed that the presence of COL in the nanocomposite increased the hemostatic effect and wound healing, and the presence of CS gave the nanocomposite high antibacterial properties. Recently, the use of inorganic antibacterial agents such as metal nanoparticles or metal oxides, nanosilicates, etc. in combination with BC as new antibacterial agents has been investigated and researched (Subramaniam and Girish, 2020). In a study, BC films were prepared with oxidized 2,2,6,6-tetramethylpiperidinyloxy (TEMPO) (TOBCP) and then combined with silver nanoparticles (Ag NPs) (diameter 16.5 nm) by thermal reduction method without reducing agent. The results of the experiments showed that the TOBCP/Ag NPs composite has excellent antibacterial activity against *E. coli* and *S. aureus* and favorable biocompatibility as wound dressings (Wu et al., 2018a). In another study, BC/Ag NPs films were prepared using the *in situ* immersion method in the presence of sodium tripolyphosphate. Examining the antibacterial properties of the film showed that its antibacterial activity against *E. coli* and *S. aureus* was 100% and 99.99%, respectively. Also, due to the transparency of the film, the wound can be evaluated and observed without removing the dressing (Tabaii and Emtiazi, 2018). Adding antibiotics to BC is the most commonly used method to prepare BC-based composites and increase their antibacterial properties. The most commonly used antibiotics are ceftriaxone, ciprofloxacin, amoxicillin, and tetracycline hydrochloride (TCH). For example, in a study, BC composite loaded with ceftriaxone and amikacin was prepared by immersion method in antibiotic solution. The evaluation results showed that BC/Ceftriaxone/Amikacin composite shows high antibacterial activity against various bacteria (such as *E. coli, S. aureus*, *Pseudomonas aeruginosa*, etc.) (Volova et al., 2018). In another study, Ye et al. prepared a new sponge with high biocompatibility and excellent antibacterial properties by grafting amoxicillin onto regenerated bacterial cellulose (RBC). The results showed that the presence of RBC increased the antibacterial activity of the sponge against bacteria and fungi, which can be used as a suitable option in the application of wound healing (Ye et al., 2018).

#### 3.1.5 Hyaluronic acid-based wound dressings

**FIGURE 7:**
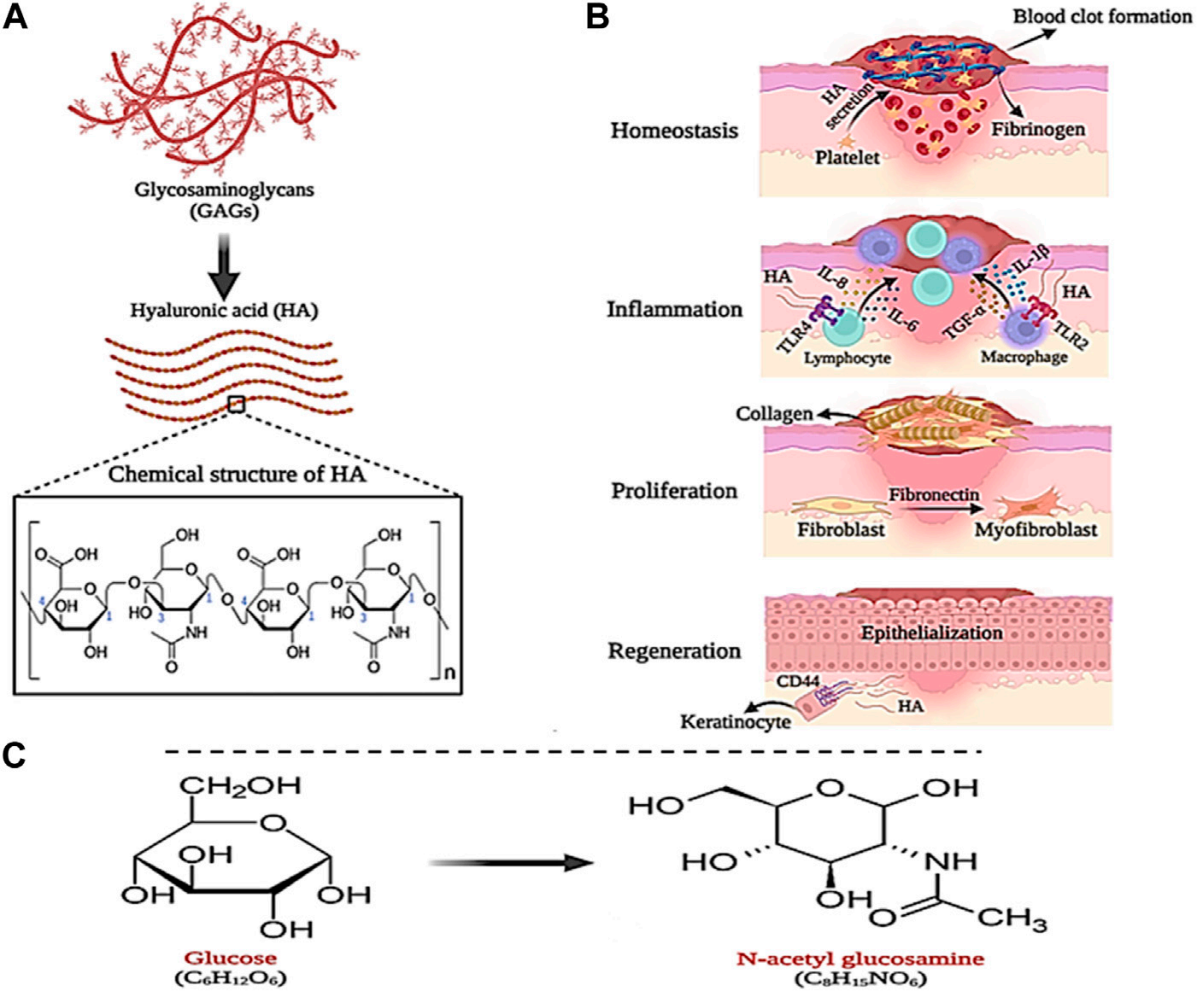
Schematic illustration of; **(A)** HA structure, **(B)** its role in wound healing and **(C)** GlcNAc chemical structure. Created with BioRender.com.

Hyaluronic acid (HA) is a natural polymer that is a subset of a group of glycans called glycosaminoglycans (GAG). Glycosaminoglycans (GAGs) are a subset of heteropolysaccharides. HA is usually present in tissues such as the umbilical cord, skin, vitreous, corolla, joints, and connective tissues. Also, HA can be produced from microbial fermentation (Liu et al., 2011; Fallacara et al., 2018; Gupta et al., 2019). HA consists of repeating units of linear polysaccharide 2-acetamido-2-deoxy-D-glucopyranose linked through β-(1–3) and D-glucopyranuronic acid linked to β-(1–4), which has a negative charge (anionic) (Figures 7A). HA can be composed of 25,000 (or more) disaccharide units, giving rise to polymers of varying molecular weight (Khan et al., 2013; Li et al., 2020). The molecular weight (MW) of HA can be in the range of 10–6,000 kDa, where HA with a MW less than 10 kDa (MW < 10 kDa) forms HA oligosaccharides (O-HA). MW between 10–25 kDa leads to the formation of low molecular weight HA (LMW-HA). Also, MW between 25–100 kDa produces medium molecular weight HA (MMW-HA). MWs higher than 100 and 600 kDa also produce high molecular weight HA (HMW-HA) and very high molecular weight HA (vHMW-HA), respectively (Khan et al., 2013). Due to the presence of N-acetyl, acetamide, and carboxylic functional groups in the molecular structure of HA, it has high water absorption and retention properties (0.5 g/L) (Tavianatou et al., 2019). However, the negative (anionic) charge of HA carboxylic groups can be balanced under physiological conditions by positively charged (cationic) ions such as Ca^2+^, Mg^2+^, Na^+^, and K^+^. This action may increase moisture absorption, biocompatibility, as well as tissue flexibility, and viscoelasticity (Lapcık et al., 1998; Fallacara et al., 2018; Gupta et al., 2019). Furthermore, HA is sensitive to pH changes and hydrolyzes under strongly acidic or alkaline conditions (pH between 4 and 11), which gives it unique and defining properties that can be used in biomedical applications (such as joint stabilizers, sutures, etc.), wound dressings, drug delivery, and cosmetics (Kavasi et al., 2017). The synthesis of HA in the biological environment is carried out by hyaluronan synthase (HAS). In the mammalian body, there are three types of HAS, namely, HAS-1, HAS-2, and HAS-3, which can produce HA of different molecular weights (Lapcık et al., 1998). Therefore, the molecular weight of HA is considered an influential factor in biological processes. It has been shown that HMW-HA has the ability to induce immune and inflammatory responses in the body, while LMW-HA can affect enzyme activity (Fallacara et al., 2018). In addition, HA can participate in the wound healing process, which is why it can be considered a suitable candidate for wound dressing.

As mentioned in the previous section, the wound healing process begins quickly after causing damage to the skin tissue in four stages, which leads to stopping the bleeding and regeneration of the skin tissue. In this process, HA plays a significant role. During the hemostasis phase, a large amount of HA is secreted from platelets, which causes fibrinogen deposition and the formation of a primary blood clot (Khan et al., 2013). Likewise, HA directs the recruitment of neutrophils involved in phagocytosis and removal of dead cells, and secretion of interleukins IL-8 and IL-1β as well as α-TNF (Maleki et al., 2008). In the last stage of the inflammatory phase, macrophages and lymphocytes migrate to the wound site, where HA (LMW-HA) communicates with their Toll-like receptors (TLR4 and TLR2) and stimulates the expression of interleukins IL-1β, IL- 6, and IL-8 and α-TNF. In addition, with the help of fibronectin, LMW-HA increases the migration, proliferation, and differentiation of fibroblast cells into myofibroblasts, which play a central role in wound healing and cause collagen deposition on the wound (Wolny et al., 2010; Aya and Stern, 2014). Finally, in the tissue regeneration phase, HA (LMW-HA) interacts with CD44 receptors on the keratinocyte cell surface and stimulates and regulates the epithelialization process (Figures 7B) (Leng et al., 2019).

HA-based wound dressings due to the various properties of this polysaccharide, such as suitable biodegradability, high biocompatibility and non-toxicity, flexibility and viscoelasticity, hydrophilicity and excellent water absorption, are available in various forms such as hydrogels, sponges, scaffolds and films (Longinotti, 2014; Mahedia et al., 2016; Zamboni et al., 2018). For example, in one study, porous sponges were prepared by combining chitosan (CS), alginate (ALG), and HA (MW = 417 Da) by freeze-drying method. Examining the morphology of CS/ALG/HA sponges by scanning electron microscope (SEM) showed that these sponges show higher porosity compared to CS/ALG sponges (without HA). Also, after incubation of sponges with HA, the rate of nutrient transport, O_2_, and cell proliferation at the damaged site increased (Orellana et al., 2016). In another study, sodium carboxymethyl cellulose (CMC-Na) was prepared by combining adipic dihydrazide (ADH) with HA and HA-CMCNa sponge. The results showed that with the increase in the concentration of ADH and 1-ethyl-3-[3-(dimethylaminopropyl)] in the sponge, the rate of degradation decreased. This high stability in HA-CMCNa sponges was shown to be successful in skin regeneration applications (Liu et al., 2007). In one study, HA-based films grafted with pullulan (Pu) (HA-g-Pu) were produced to increase the biological performance and stability of HA in the wound healing process. The results of the investigations showed that the HA-g-Pu film presents a higher swelling ratio (40, 30, and 34%, respectively) compared to the pure Pu and HA films. Also, it was shown that HA-g-Pu films were completely degraded after 12–14 days, while HA film alone was completely degraded ±3 days after incubation. Therefore, it was confirmed that the binding of Pu to HA increases the stability of HA-based films. In addition, SEM images showed the porosity of HA-g-Pu films between 29.36 ± 73.13 μm, which is suitable and compatible for cell migration. Finally, the test results showed that HA-g-Pu films exhibit high stability and excellent biocompatibility, which can be useful in accelerating the wound process (Li et al., 2018). In research, cross-linked HA-hyaluronic-(2-aminoethyl)-carbamate-α-elastin (HA-EDA) hydrogels were prepared to release vascular endothelial growth factor (VEGF) in the wound environment. Evaluation of the release of VEGF from the hydrogel showed that after 5 days of incubation, 50% of VEGF was retained in the hydrogel, which stimulated the proliferation of human vascular endothelial cells (HUVEC) (which is effective in the angiogenesis process during wound healing) (Fiorica et al., 2018).

#### 3.1.6 N-acetylglucosamine-based wound dressings

N-acetylglucosamine (GlcNAc) is an amino monosaccharide with the chemical structure C_8_H_15_NO_6_, which is derived from glucose and is also known as 2-(acetylamino)-2-deoxy-D-glucose or acetamino-2-deoxy-β-D-glucose (Figures 7C) (Umekawa et al., 2022). This amino monosaccharide is found as a white and sweet powder with a melting temperature of 221°C. GlcNAc is soluble in water and produces clear and colorless aqueous solutions (Du et al., 2022). Toxicity tests have shown that GlcNAc does not cause any toxicity and the half-life of this amino monosaccharide after subcutaneous injection of 20 g of it has been reported to be 220 min (Kaga et al., 2020).

It has been reported in studies that GlcNAc, as the main part of epithelial, plays an essential role in wound healing. It has been shown that the injection of GlcNAc can increase the amount of HA production in surgical wounds and reduce the occurrence of side effects in the tissue repair process (Shi et al., 2018; Schoukens, 2019; Prakashan et al., 2023). For example, in a study, the effect of local injection of GlcNAc on the wound healing process in rat was investigated. The results showed that after GlcNAc injection, collagen synthesis and fibroblast proliferation increased and improved the wound angiogenesis process. Moreover, it was shown that the rate of wound closure was favorably increased (Ashkani-Esfahani et al., 2012).

As mentioned, GlcNAc plays an essential role in wound healing. It has been proven that GlcNAc is found in the block structures of connective tissues (such as proteoglycans, glycoproteins, and glycosaminoglycans (GAGs), which are effective as the main substrate in anti-inflammatory reactions and repair of damaged tissue (Ganesh et al., 2023; Segars and Trinkaus-Randall, 2023). Also, GlcNAc increases the production and proliferation of fibroblasts, HA, and keratinocytes in the skin, which can be used as a suitable option for wound healing (Minami and Okamoto, 2007; Wang et al., 2021). For example, in one study, chitosan (CS) and N-acetyl-D-glucosamine were used to produce absorbable sutures in post-surgical wound repair. In this study, chitosan fibers were combined with N-acetyl-D-glucosamine by wet spinning method and their mechanical and biological properties were investigated. The results showed that the mechanical resistance decreased after adding GlcNAc to CS, however, this value was still higher than the average value declared by the US Food and Drug Administration (FDA) for #6–0 sutures (1.7 N). Furthermore, the cytotoxicity of pure CS and CS/GlcNAc threads was evaluated on L929 cells. The results showed no cytotoxicity for CS/GlcNAc. In addition, CS/GlcNAc sutures, due to the long-term release of GlcNAc, caused pain relief, reduced surgical infection, and finally improved the surgical wound healing process. Therefore, CS/GlcNAc produced sutures can be introduced as a suitable option in the preparation of absorbable sutures in surgical wound repair due to their excellent biocompatibility, favorable biodegradability, and long-term release of GlcNAc drug (da Silva et al., 2019). In another study, the effect of poly-N-acetylglucosamine (sNAG) suspensions on the healing of leg and leg venous wounds and damaged tendons was investigated in a rat model. The results showed that sNAG increased the tangential stiffness and quasi-static stiffness of the tendon during the fatigue cycle. Also, sNAG increased the dynamic and viscoelastic modulus of the tendon. Furthermore, sNAG did not show any negative effects in the toxicity test (non-toxicity). According to these results, sNAG can be studied as a non-invasive and therapeutic method for tendon wound repair (Nuss et al., 2021). Scherer et al. developed a bioactive scaffold based on N-acetylglucosamine (sNAG) for the treatment of diabetic wounds. The results showed that sNAG scaffolds stimulate the migration of fibroblast and endothelial cells. Also, compared to the control group, the sNAG scaffold increased the rate of cell proliferation up to 4 times, the angiogenesis processes up to 2.7 times, the migration of keratinocytes up to 7.5 times, and the formation of granular tissue up to 2.8 times. Finally, it was concluded that the sNAG bioactive scaffold can be useful for the healing of diabetic wounds due to its favorable effect on the process of angiogenesis and re-epithelialization (Scherer et al., 2009).

#### 3.1.7 Collagen-based wound dressings

**FIGURE 8:**
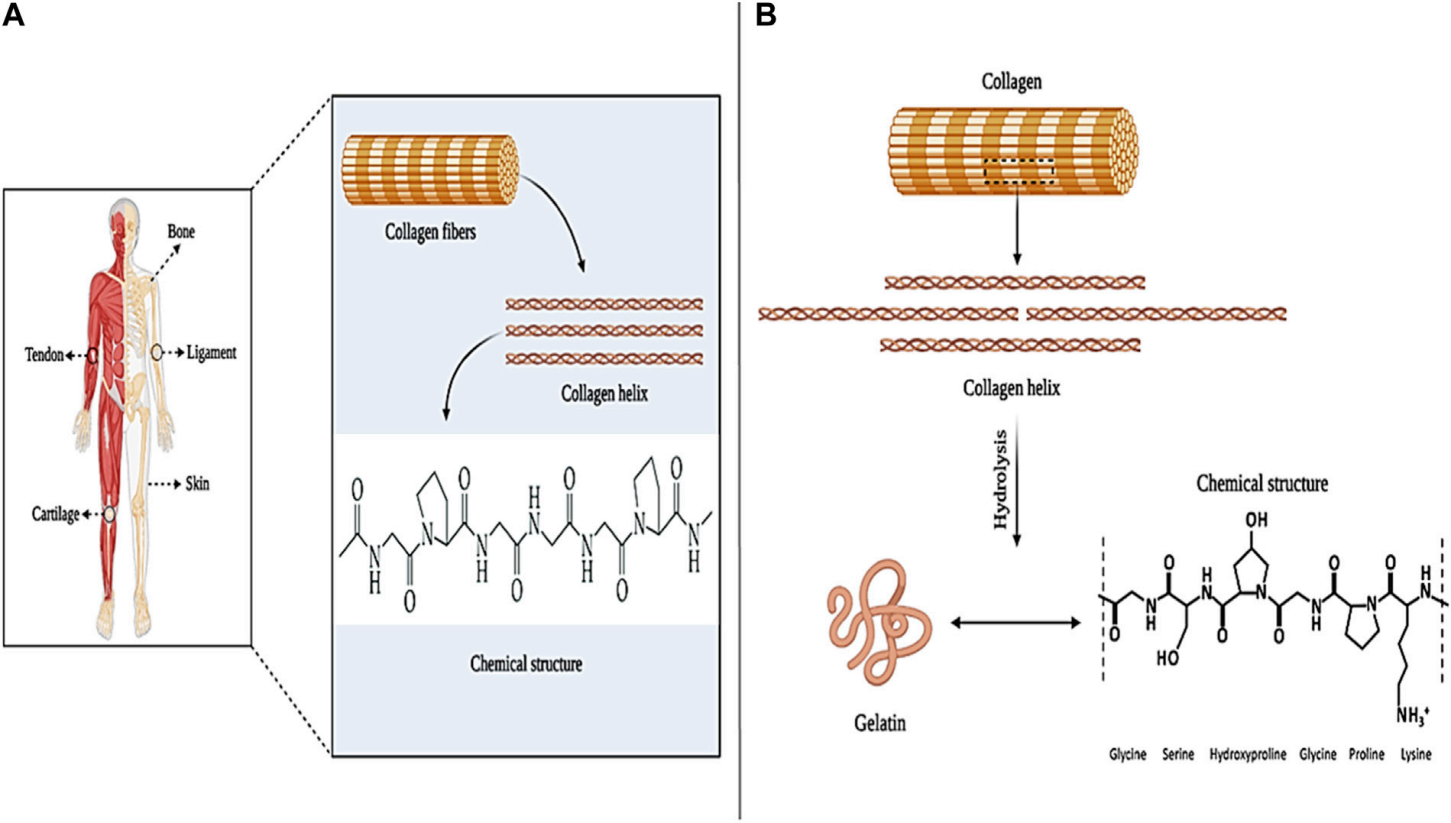
Collagen panel; **(A)** collagen sources and chemical structure, and **(B)** gelatin chemical structure. Created with BioRender.com.

Collagen is the most common and abundant protein in the human body, which is found in various tissues such as skin, cartilage, tendons, ligaments, and bones. So far, 29 different types of collagens (such as type I, II, and III) have been identified in the body (Figures 8A) (Parenteau-Bareil et al., 2010). The most common type of collagen in the human body is collagen type I, which constitutes between 70% and 80% of collagens in the body and is mostly found in the form of intertwined fibers in skin, tendon, and bone tissue. After type I collagen, type VI, XII, XIV, and XVI pseudofibrillar collagens are the most abundant in the skin. In addition, type IV and XVII collagens with a non-fibrillar structure are present in the skin membrane (Wenzel et al., 2006; Gould, 2016; Nijhuis et al., 2019). Collagen, as a structural component of ECM, affects cell proliferation and differentiation in the wound healing process. After an injury, collagen stops bleeding by activating the blood clot cascade and creating a fibrin clot. Also, type I and IV collagens by attracting neutrophils lead to increased immune response and phagocytosis, which are known as inflammatory mediators (Ricard-Blum, 2011; Ricard-Blum and Ballut, 2011). In addition, it has been shown that collagen (especially collagen type I) is effective in the role of inhibitor or stimulator in the process of angiogenesis. For example, collagen type I, through its C-propeptide fragments, induces the recruitment of endothelial cells, induces angiogenesis, and ultimately regenerates the ECM in the wound (Kisling et al., 2019). In contrast, fragments of proteolytic type IV and XVIII collagens (such as arrestin, thomasstatin, constatin, and endostatin) have been shown to act as inhibitors of endothelial cell proliferation and migration and induce endothelial cell apoptosis (Kareva et al., 2016).

Collagen, due to the favorable properties of biocompatibility, biodegradability, low immunogenicity, degradability by enzymes, non-toxicity, availability, etc., which are all effective in cell proliferation and adhesion, can be used in various applications such as wound healing, hemostatic, bone tissue engineering and ophthalmology (Ferreira et al., 2012; Abou Neel et al., 2013). For example, in one study collagen/poly D-L-lactide-glycolide (PLGA)-based scaffolds were prepared and loaded with the drug Glucophage (a type of anti-diabetic drug). The results of the evaluations showed that the collagen/PLGA scaffold increased collagen production and epithelialization of the skin tissue in the rat model with diabetic wounds (Lee et al., 2015). In another study, a composite hydrogel based on bacterial cellulose/collagen (BC/COL) was prepared for wound healing application. The results of the *in vivo* experiments showed that the BC/COL hydrogel on the seventh day after implantation in the back of the mouse caused the repair of the damaged tissue. Also, BC/COL hydrogel showed better collagen fiber orientation (*p* = 0.0001) compared to collagenase ointment and control groups (Moraes et al., 2016). In addition, Ansari et al. (2018a) produced a collagen/glycosaminoglycan (COL/GAG)-based cellular scaffold for use in wound healing. Cell evaluation after implantation of amniotic fluid-derived stem cells (AFS) on COL/GAG scaffolds showed that the produced scaffolds increase the adhesion and proliferation of AFS cells, which is recognized as an attractive cell source in wound healing and skin tissue regeneration. Therefore, according to the obtained results, collagen-based repairers can be used as desirable wound dressings in skin tissue regeneration and wound healing applications.

#### 3.1.8 Gelatin-based wound dressings

Gelatin (Gel) is a natural polymer derived from collagen, which is available in two common types, A and B (Figure 8B). Type A gelatin is produced through the hydrolysis of insoluble collagen in an acidic medium and has 18.5% nitrogen (N_2_) in its structure (Nikkhah et al., 2016). Type B gelatin is obtained through the alkalization of collagen (due to the lack of amide functional groups) and has only 18% N_2_ in its structure (Shi et al., 2016). Gelatin, in terms of composition, is similar to collagen and consists of glycine, proline, and hydroxyproline. Also, since gelatin is derived from collagen, it has almost the same characteristics as it, such as; Biocompatibility, biodegradability, biomimicry of ECM, flexibility, stability, homeostatic effect, etc. These properties make collagen a desirable option in wound healing, bone tissue engineering, gene delivery, and drug delivery applications (Nair and Laurencin, 2006; Nichol et al., 2010; Parvez et al., 2012; Sulaiman et al., 2020).

From the past until now, many studies have been conducted regarding the characteristics and applications of gelatin as wound healer. For example, in a study, nanofibers based on gelatin and oxidized sucrose were prepared by the electrospinning method. Evaluation of the toxicity of oxidized gelatin/sucrose nanofibers on L-929 cells showed that these nanofibers do not show any toxicity and have good biocompatibility (Jalaja and James, 2015). In another study, horseradish peroxidase (HRP)-catalyzed gelatin-based hydrogels were prepared that could be sprayed on a diabetic wound and the cytokines MIP-3a and IL-8 were loaded *in situ*. Then, these hydrogels were evaluated in a streptozotocin (STZ)-induced diabetic mouse model. The results showed that gelatin/HRP hydrogel improves the process of neovascularization and tissue granulation. Also, it can lead to faster wound closure (Yoon et al., 2016). Mao et al. developed a shape-memory hydrogel based on oxidized starch and gelatin (OSG) to improve wound closure non-invasively. The investigation of OSG hydrogel after implantation on the wound of a rabbit model showed that OSG hydrogel causes the formation of thick layers of dermis and epidermis in the rabbit model and improves tissue regeneration. Also, no scar was observed on the skin of rabbits treated with OSG compared to the control group. These results showed that OSG hydrogel can be introduced as a suitable alternative to sutures for wound closure (Mao et al., 2020). The effects of natural gelatin-based nanocomposites on cell adhesion and wound healing were investigated in an *in vivo* study by Nikpasand and Parvizi (2019) The results of the evaluations showed that natural gelatin promotes the survival and adhesion of fibroblast cells between 72 h and 7 days. In addition, natural gelatin increased angiogenesis, tissue granulation, and re-epithelialization without causing toxicity. These results show that gelatin as a biocompatible, biodegradable, flexible, hydrophilic, gas barrier, and hemostatic natural biomaterial can be studied and used in wound healing and skin tissue engineering applications.

#### 3.1.9 Dextran-based wound dressings

**FIGURE 9:**
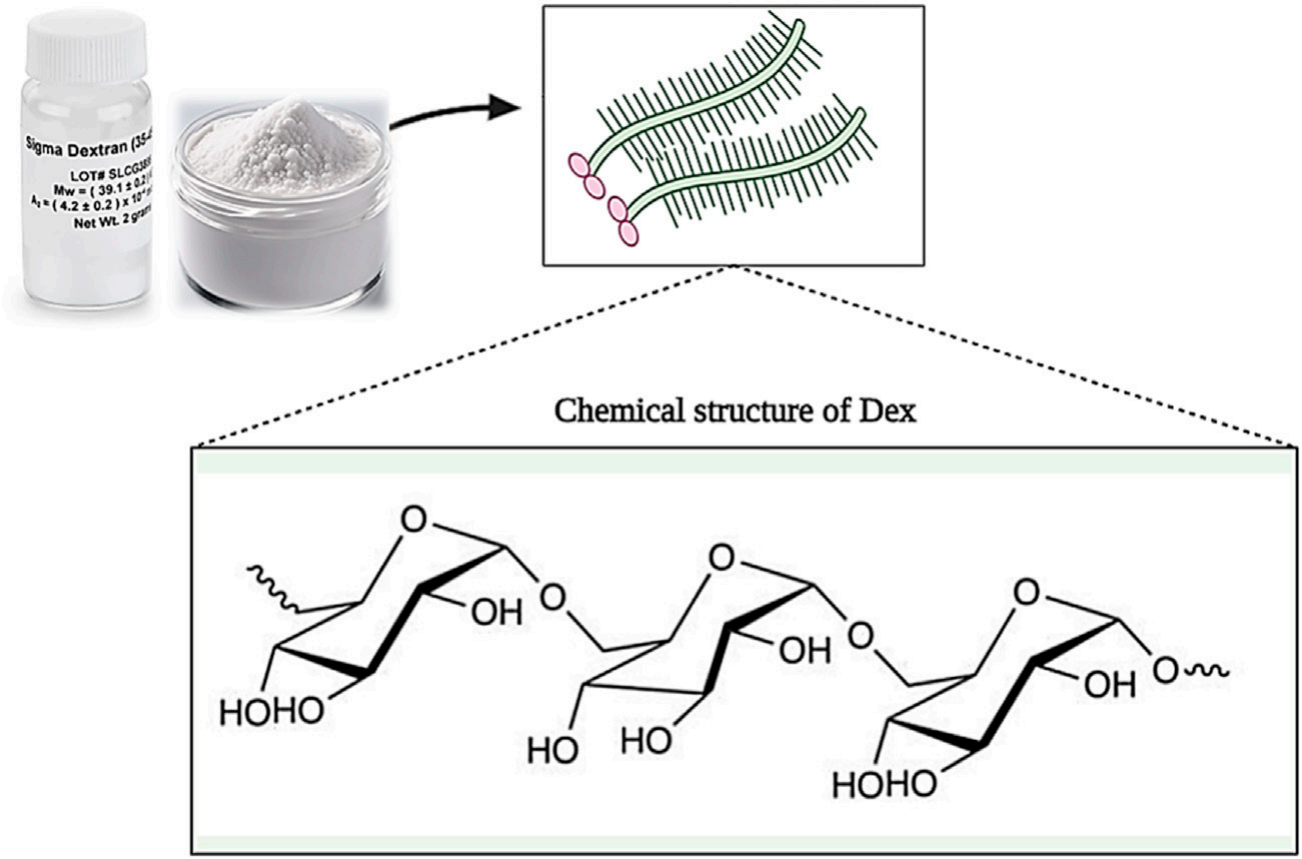
Schematic illustration of the chemical structure of Dextran. Created with BioRender.com.

Dextran (Dex) is a group of microbial glucans that is formed from linear D-glucose monomers linkage with α-(1–6), α-(1–2), α-(1–3), and α-(1–4) (Figure 9). Moreover, dextran can be produced using sucrose. In this case, Leuconostoc mesenteroides NRRL B-512F and Leuconostoc mesenteroides NRRL B-1299 are commonly used (Huang and Huang, 2018). Different types of dextran show different physicochemical and biological properties. Medical grade dextran has a molecular weight between 40 and 70 kDa and has high thermal resistance in the sterilization process. Low molecular weight dextran is obtained through acid hydrolysis of high molecular weight dextran, which acts as a facilitator of blood flow and prevents the accumulation of red blood cells. Also, by changing the concentration of sucrose in the synthesis process, dextran with different molecular weights can be produced (Varshosaz, 2012; Ghimici and Nichifor, 2018). Dextran has the ability to dissolve in various solvents such as water, ethylene glycol, and dimethyl sulfoxide (DMSO). In addition, dextran is decomposed by enzymes in the biological environment, and its products are removed from the environment over time without causing toxicity (Hoang et al., 2021).

It has been shown that dextran is effective in the wound healing process in various ways. For example, dextran can play the role of an artificial medium for cell culture, proliferation, and differentiation. Also, it can prevent ischemic damage to the skin and strengthen and increase the angiogenesis process. In addition, dextran also affects the formation of granulation tissue and can improve tissue regeneration and wound healing by inducing collagen deposition (Sun et al., 2011; Wang et al., 2019b; Khan et al., 2019). Many studies have been conducted on the use of dextran as a wound healer. For example, in one study, hydrogels based on oxidized dextran/hyaluronic hydrazide were prepared for wound healing and loaded with brain-derived neurotrophic factors. The results showed that hydrogels loaded with neurotrophic factors, unlike pure hydrogels, increase cell proliferation and wound healing (Huang et al., 2021). In another study, a dextran allyl isocyanate ethylamine (Dex-AE)/polyethylene glycol diacrylate (PEGDA) based hydrogel was produced. The results of evaluations (during 3 weeks) showed that hydrogel (Dex-AE/PEGDA) with a monomer ratio of 80:20 increases neovascularization, and tissue penetration and stimulates skin regeneration (Sun et al., 2011). Wang et al. produced a natural hydrogel based on Dex, HA, and β-cyclodextrin (β-CD) and loaded a combination of VEGF-expressing plasmid and resveratrol (Res) genes into it. Observations showed that hydrogel (Gel-Res/pDNAVEGF) increased biocompatibility, reduced wound inflammation, increased microvascular growth, and ultimately burn wound healing (Wang et al., 2019a).

Therefore, according to the studies carried out, it can be said that dextran has the potential to be used as a suitable and promising wound healer due to its interesting characteristics such as hydrophilicity, biodegradability, biocompatibility, non-toxicity, antioxidant, degradability by enzymes, etc.

#### 3.1.10 Chitin/chitosan-based wound dressings

**FIGURE 10:**
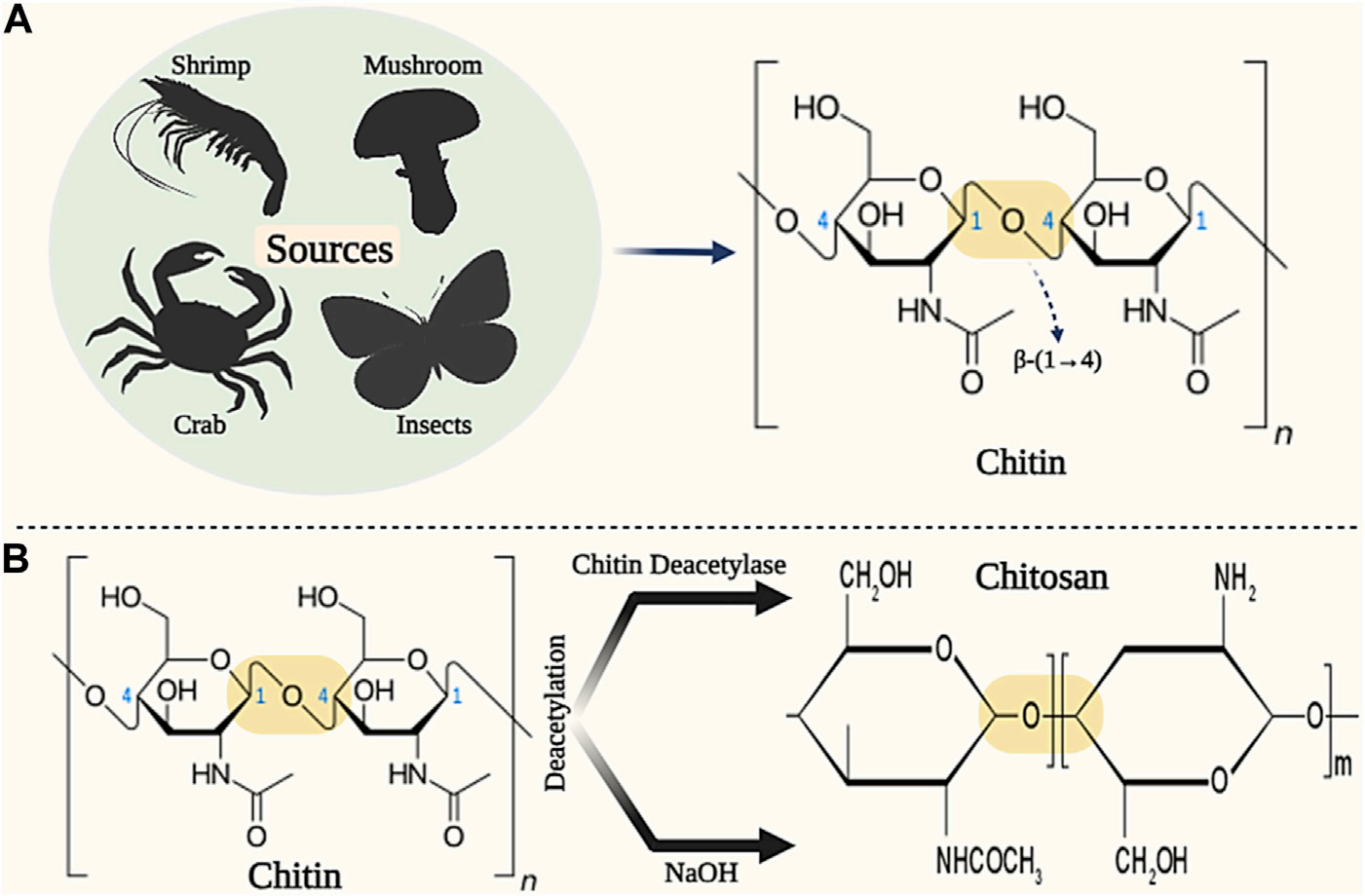
Schematic illustration of the sources and chemical structure of **(A)** Chitin and **(B)** Chitosan. Created with BioRender.com.

Chitin with long hydrophobic linear chains is known as the most abundant animal polysaccharide, consisting of 2-acetamido-2-deoxy-D-glucose units linked by β-(1–4) bonds (Figures 10A) (Aravamudhan et al., 2014). Chitin is found in various sources such as insects, crabs, shrimps, and mushrooms. This natural biopolymer is insoluble in many solvents (such as water and other organic solvents), but it shows good solubility in solvents such as dimethylacetamide with 5% lithium chloride, chloro-alcohols and hexafluoro-isopropanol. Chitin, unlike other natural biopolymers, exhibits alkaline properties, which gives it gelling properties for various applications (Jayakumar et al., 2010; Abo Elsoud and El Kady, 2019).

Chitosan (CS) is a semi-crystalline polysaccharide with a cationic charge, which consists of monomeric units of N-acetylglucosamine and glucosamine linked by β-(1–4) bond (Figures 10B) (Singh et al., 2017b). Chitosan is produced through deacetylation or deacetylation of glucosamine chitin units (degree of acetylation or deacetylation higher than 50%). Deacetylation of chitin is done through the enzyme chitin deacetylase and deacetylation of chitin is done by alkaline solutions (such as NaOH) (Zhu et al., 2019). The molecular weight of chitosan varies between 300 and 1,000 kDa, depending on the production method and the degree of deacetylation (between 60% and 95%), which makes it suitable for diverse applications (Zhang et al., 2023).

Chitin and chitosan have desirable biological properties such as biodegradability, biocompatibility, degradable by lysozyme enzyme, antimicrobial, non-allergic, non-toxic, and hemostatic effect, which makes them suitable for various applications such as wound healing (Kean and Thanou, 2010; Ibitoye et al., 2018; Baharlouei and Rahman, 2022; Islam et al., 2022; Sheng et al., 2022). Chitin has been shown to affect red blood cell flow, vasoconstriction, platelets, and blood clotting factors at the site of injury (Liu et al., 2018). Also, chitosan has shown the ability to connect, stick, and multiply cells properly. In addition, it has been shown in studies that chitin and its derivatives show antibacterial and analgesic properties that stimulate tissue regeneration and accelerate wound healing (Singh et al., 2017c; Zhao et al., 2020). Hence, chitin and chitosan in different forms (such as films, hydrogels, and fibers) have been used as suitable candidates for wound dressing applications (Naseri-Nosar and Ziora, 2018; Elangwe et al., 2023). For example, in one study, chitosan (CS)/polyacrylic acid (PAA) based hybrid nanoparticles (CS-PAAhybrid NPs) were prepared by free radical polymerization (FRP) method for post-surgical wound healing applications. Examining the physicochemical properties of CS-PAAhybrid NPs showed that mechanical strength and resistance were 50–120 kPa for 1:1 Cs/AA ratios and 120–230 kPa for 1:0.2 Cs/AA ratios. Characterization of cytotoxicity on CS-PAAhybrid NPs also showed good cell biocompatibility (cell activity higher than 75%). Furthermore, the hemolytic test of CS-PAAhybrid NPs samples showed less than 0.5% damage (according to the standard) on red blood cells. Finally, the researchers concluded that CS-PAAhybrid NPs can be used as a desirable wound dressing in wound healing applications (Saberi et al., 2018). In another study, chitosan and gelatin were immobilized on the surface of silicone/acrylic acid films for the application of skin tissue engineering and wound healing. The results of the evaluations showed that with the increase in the concentration of chitosan and acrylic acid, the hydrophilicity of the film surface also increases. Also, the adhesion, growth and proliferation of L-929 cells on the films modified with chitosan and gelatin increased compared to the pure film. Therefore, the researchers found the produced product suitable for burn wound healing applications due to its excellent biocompatibility (Ansari et al., 2018b). In a research study, the effect of chitosan-based gels on 2^nd^-degree burn wounds was investigated. In this study, first-grade 2 burns were created on 6 rabbit models. Then, rabbits were treated using chitosan gels. The results showed that chitosan gel increased the speed of re-epithelialization, reduced scarring, improved skin color, and ultimately reduced the duration of wound healing (Ansari, 2020). In two similar studies recently conducted by Ansari et al., the effect of chitosan-based hydrogels on burn wound healing was characterized (Ansari et al., 2022; Ansari et al., 2023). In the first study, first, full-thickness burns were created on the back area of Wistar albino model mice. Then, mice were treated with chitosan gel, chitosan/aloe vera hydrogel, chitosan/guar gum hydrogel, and chitosan/guar gum/aloe vera hydrogel (CS/GG/AV) respectively. The results showed that CS/GG/AV hydrogel improved angiogenesis, epithelial regeneration, and overall wound healing compared to the rest of the groups. Also, CS/GG/AV hydrogel did not induce inflammatory responses during the wound healing process. As a result, CS/GG/AV hydrogel was introduced as a desirable wound dressing for burn wounds (Ansari et al., 2022). In another study, Ansari et al. (2023) investigated the effect of chitosan/guar gum/peppermint essential oil (CS/GG/PEO) antibacterial hydrogel on the healing of burn wound infection in mice. Histopathological evaluation results showed that CS/GG/PEO hydrogel significantly improves the angiogenesis process, collagen fiber thickness, re-epithelialization, and wound contraction (90% on day 22). Therefore, chitin/chitosan can be considered ideal wound dressings due to having suitable physicochemical and biological properties.

#### 3.1.11 β-Glucan-based wound dressings

**FIGURE 11:**
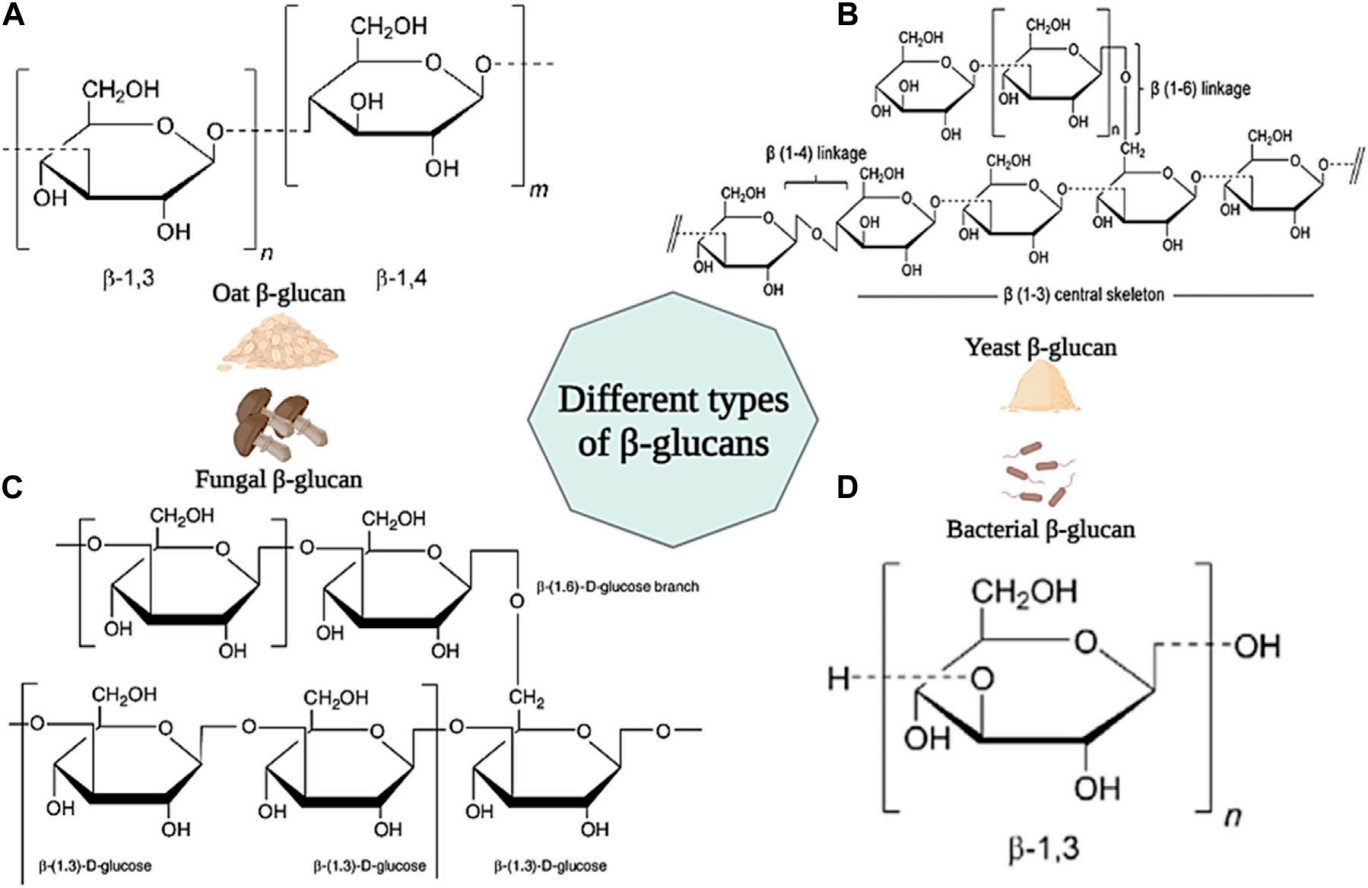
Schematic illustration of different type of β-glucans and their chemical structures. **(A)** β-glucan from Oat; **(B)** β-glucan from Yeast; **(C)** β-glucan from Fungal; **(D)** β-glucan from Bacteria. Created with BioRender.com.

β-Glucan is a group of carbohydrate polysaccharides consisting of d-glucose units linked by β-(1–3), β-(1–4) and β-(1–6) bonds. β-Glucan is found in the cell walls of many microorganisms such as fungi, bacteria, yeasts, and cereals (such as oats) (Lattimer and Haub, 2010). Generally, all beta-glucans have d-glucose units linked by β-(1–3) bonds in their structure. However, beta-glucan found in cereals has both β-(1–3) and β-(1–4) linkages in its backbone. Beta-glucan found in fungi and yeasts have short β-(1–6) and long β-(1–6) linkage branches in their structure, respectively. However, β-glucan in bacteria has a linear structure and no branches in its backbone (Figures 11A–D) (Volman et al., 2008; Wani et al., 2021).

Different β-glucans show a variety of physicochemical properties (such as solubility, molecular weight, and viscosity) that will also affect their biological properties (Seo et al., 2019). It has been shown in studies that β-glucan is effective in reducing blood cholesterol, and blood pressure, the antagonist of benign and malignant tumors due to its anticancer, antibiotic, and strong immune-stimulating properties (Bashir and Choi, 2017). Also, β-glucan has the ability to stimulate the production of growth factors necessary for skin repair, adjust the moisture and elasticity of the skin, and increase collagen biosynthesis. In addition, it has been shown that β-glucan exhibits antibacterial and antimicrobial properties against various types of bacteria such as *S. aureus*, *E. coli*, *P. aeruginosa*, etc. Also, by increasing phagocytosis, it increases resistance to microbial agents (Chinnu et al., 2014; Majtan and Jesenak, 2018). For the first time, Leibovich and Danon used β-glucan for wound healing in 1980. The results of their experiments showed that β-glucan stimulates wound healing and accelerates epithelialization by increasing the activity of macrophages and decreasing the number of polymorphonuclear neutrophils (Leibovich and Danon, 1980). In another study, β-glucan in barley was shown to stimulate cell proliferation and accelerate wound closure by stimulating human dermal fibroblast (HDF) cells (Fusté et al., 2019). Van den Berg et al. confirmed the effect of β-glucan on increasing wound immunity and burn wound healing (van den Berg et al., 2014). In another study, the effects of four types of β-glucans derived from mushrooms, yeast, barley, and euglena on wound healing were investigated. The results showed that all β-glucans caused the migration of keratinocytes and wound closure. Also, none of the β-glucans showed a toxic effect on skin fibroblast cells (Seo et al., 2019). In a clinical study, the effect of yeast β-glucan ointment on cytokines and second and third-degree burn wounds was investigated. 33 patients received 5% yeast β-glucan ointment and Stratamed ointment in two groups of 23 and 10, respectively. The evaluation results showed that patients who received β-glucan ointment showed better wound healing (RR = 4.34; 95% Cl; 0.73 to 25.67; *p* = 0.11). In addition, patients receiving β-glucan ointment showed a significant difference in the level of IL-4 cytokine secretion compared to the control group (Abedini et al., 2022). According to these results, β-glucan can be mentioned as an ideal natural biopolymer in wound healing applications.

### 3.2 Synthetic biomaterials in wound healing

Synthetic biomaterials have been used in many applications such as hard and soft tissue engineering, wound healing, and drug delivery due to their bioabsorbability, biocompatibility, low toxicity, controlled synthesis and modification, biodegradability, and compatibility with intended use (Table 2) (Mir et al., 2018; Prete et al., 2023). Some common synthetic biomaterials that have been used in wound healing include polyglycolic acid (PGA), polyvinyl alcohol (PVA), polyethylene glycol (PEG), polyethylene oxide (PEO), and polyvinyl pyrrolidone (PVP) (Qiu and Bae, 2006). In the following, we will briefly review these biomaterials and their applications in wound healing.

**TABLE 2:** Structure and properties of synthetic biomaterials used in wound healing.

Synthetic biomaterials	Structure	Properties	References
PGA	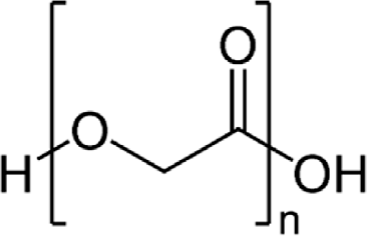	strong absorbent, thermoplastic, biodegradability, low toxicity, biocompatibility, and preventing the penetration of gases	Yamane et al. (2014), Zha et al. (2022)
PVA	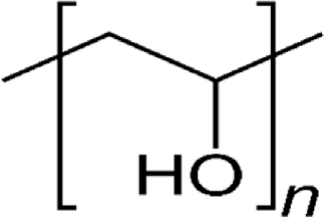	excellent mechanical properties, high solubility, low toxicity, biodegradability, and biocompatibility	Pan et al. (2019), Radulescu et al. (2022)
PEG	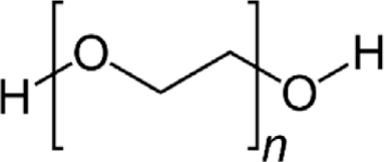	hydrophobicity, biodegradability, non-toxicity, non-immunogenicity, and biocompatibility	Shi et al. (2022)
PEO	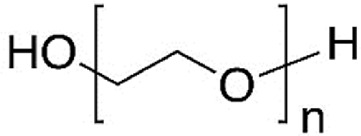	non-ionic polymer, viscoelasticity, water solubility, biodegradability, and biocompatibility	Yamaguchi et al. (2015), Govindasamy et al. (2020)
PVP	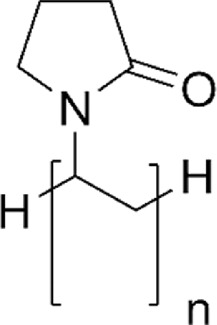	biocompatibility, biodegradability, antibacterial, water-soluble, heat-resistant, high wettability, and low toxicity	Contardi et al. (2021)

#### 3.2.1 Polyglycolic acid (PGA)

Polyglycolic acid (PGA) is a synthetic, biodegradable, thermoplastic polymer with high mechanical strength that can be obtained through the ring-opening polymerization of glycolic acid (Yamane et al., 2014). PGA has been used as a wound dressing due to its properties such as strong absorbent, preventing the penetration of gases, biocompatibility, and biodegradability. Also, it has been shown that PGA is effective in the inflammatory process and epithelial tissue formation (Zha et al., 2022). For example, in one study, fibrin/PGA glue was shown to prevent gas infiltration, wound fluid secretion, and complete closure and coverage of the wound surface in the dorsal region of mice (Kinoshita et al., 2005). In addition, the effectiveness of PGA in covering wounds resulting from endoscopy and open surgery has also been proven (Kouketsu et al., 2021). Zha et al. SF/PGA-based biodegradable nanofibrous scaffolds were prepared and Deferoxamine (DFO) was loaded into it as a model drug for diabetic wound healing. *In vitro* results confirmed the biocompatibility and biodegradability of the PGA/SF-DFO scaffold and showed that this scaffold provides a suitable microenvironment for cell growth, proliferation, and migration. In addition, PGA/SF-DFO scaffold led to 70% improvement in mechanical properties, re-angiogenesis, collagen deposition, and healing of diabetic wounds within 14 days (Zha et al., 2022).

#### 3.2.2 Polyvinyl alcohol (PVA)

Polyvinyl alcohol (PVA) is a synthetic and hydrophilic polymer with 3,1-diol bonds in its structure, which is formed through the hydrolysis of polyvinyl acetate. Recently, PVA has been used in biomedical and wound healing applications due to its properties such as excellent mechanical properties, high solubility, low toxicity, biodegradability, and biocompatibility (Pan et al., 2019; Radulescu et al., 2022). For example, in one study, hydrogels based on PVA/Pullulan/Poly-L-Lysine/Gelatin were prepared for wound healing applications. It was shown that these hydrogels show low toxicity and blood compatibility *in vitro* conditions. Also, hydrogels improve cell proliferation and migration in wound healing (Shitole et al., 2019). In another study, a composite hydrogel based on PVA/Dex/CS was produced as a wound dressing. The results of the evaluations showed that this hydrogel has favorable properties such as retaining water and creating a moist environment on the wound, thermal stability, good mechanical properties, and antimicrobial effect. Therefore, PVA/Dex/CS hydrogel can be used as a suitable wound dressing in wound healing (Lin et al., 2019). In research, hydrogels based on PVA/aloe vera (Av) were prepared and combined with curcumin and gentamicin. Evaluations showed that these hydrogels improve the wound healing process by affecting the re-epithelialization process and increasing it (Kenawy et al., 2023). Massarelli et al. (2021) introduced a new wound dressing based on CS/PVA with controlled release of disinfectants (chlorhexidine (CHX) and polyhexanide (PHMB)) as a safe alternative for treating wounds. Dressings containing PHMB showed appropriate properties in terms of irritation and cytotoxicity. Moreover, the antibacterial activity of dressings against *S. aureus* and S*. epidermidis* was confirmed. The use of these dressings in the treatment of dog wounds showed a faster recovery than conventional treatment.

#### 3.2.3 Polyethylene glycol (PEG)

Polyethylene glycol (PEG) is a synthetic polymer composed of ethylene oxide monomers. Also, PEG is a hydrophobic, biodegradable, non-toxic, non-immunogenic, and biocompatible polymer that is widely used in tissue engineering, drug delivery, and wound healing applications (Shi et al., 2022). Recently, PEG-based hydrogels have been investigated in the field of wound dressings due to their good biocompatibility, biodegradability, low toxicity, availability, and cost-effectiveness. However, the presence of cross-linking agents in these hydrogels may cause toxicity in wound dressing applications. To overcome this problem, citric acid (CA) has been investigated and used as a suitable cross-linking agent to reduce the toxicity of PEG-based dressings and enhance wound healing effects (Xie et al., 2015). Nevertheless, PEG is still used as a suitable synthetic biomaterial in the field of wound dressings. For example, in one study, 3D-printed scaffolds based on PEG/sodium alginate (SA) were produced and combined with Satureja cuneifolia (SC) plant extract as a model drug. The analysis of the PEG/SA/SC scaffold showed that it exhibits antibacterial properties against Gram-positive bacteria due to the presence of SC extract. In addition, the viability of L929 fibroblast cells on the scaffold was confirmed by MTT test. According to these results, the PEG/SA/SC scaffold was introduced as a promising option for diabetic wound healing (Ilhan et al., 2020). In another study, fiber mats based on PEG, poly-ε-caprolactone (PCL), and ciprofloxacin (Cip) were prepared as a wound dressing using direct-writing melt electrospinning technology. The evaluation results confirmed that the presence of PEG in this dressing improved its hydrophilicity and had a positive effect on the release of Cip drug. Furthermore, PEG/PCL-Cip composite mat showed antibacterial activity (He et al., 2019b).

#### 3.2.4 Polyethylene oxide (PEO)

Polyethylene oxide (PEO) is a non-ionic polymer that is structurally very similar to PEG. The difference between these two biopolymers is their molecular weight, the molecular weight of PEO is higher than that of PEG (Yamaguchi et al., 2015). In addition, PEO exhibits various physicochemical and biological properties such as viscoelasticity and proper lubrication, excellent water solubility, biodegradability, and biocompatibility. Also, many studies have shown that combining PEO with chitosan increases the exudate absorption capacity of wound dressings (Govindasamy et al., 2020). For example, in one study, nanofibers based on PEO and chitosan were designed for wound healing applications. The results of *in vitro* studies showed that PEO/CS nanofibers show less adhesion to the wound surface. Furthermore, they increase the exudate absorption capacity in small to medium wounds. In addition, PEO/CS nanofibers lead to increased stimulation of fibroblast cell migration in the wound healing process (Szymańska et al., 2022). Haryanto et al. Hydrogel films based on PEO/PEG dimethacrylate in different polymer concentrations were created by electron beam irradiation for wound dressing applications. The results showed that PEG MA/PEO 20% hydrogel films show the highest amount of gel fraction (76%), the highest tensile strength (0.65 MPa), and the lowest swelling ratio (235%). Based on these results, the researchers confirmed that PEG MA/PEO 20% hydrogel can be used in potential wound dressing applications (Mahardian and Fani, 2018). In another study, a composite nanofiber network of antimicrobial peptides (AMPs) and PEO was prepared by pressurized gyration method for wound healing applications and its antibacterial properties were evaluated on *S. epidermidis* species. The results of the evaluations showed that the increase of AMPs reduces the growth of bacteria. Additionally, PEO-AMP nanofibers can be designed in a way that increases the release rate of peptides. Besides, the use of pressurized gyration allows rapid mass production. According to the obtained data, researchers have introduced PEO-AMP nanofibrous networks as next-generation wound dressing approaches (Afshar et al., 2021).

#### 3.2.5 Polyvinyl pyrrolidone (PVP)

Polyvinyl pyrrolidone (PVP), as a biocompatible, biodegradable, antibacterial, water-soluble, heat-resistant biopolymer, with high wettability and adhesion and low toxicity, has recently been studied and investigated in the fields of wound healing (Contardi et al., 2021). For example, in a study, nanocomposites based on PVP/PVA and Ag/ZnO were prepared. The produced wound dressing showed suitable antibacterial properties for *S. aureus*, significantly reduced wound infection, and increased the rate of the wound healing process (Khan et al., 2021). In another study, dextran sulfate/PVP-based nanofibers loaded with the drug ciprofloxacin were designed and produced to reduce infection and wound healing. Researchers confirmed that these nanofibers exhibited remarkably high antibacterial activity against various bacteria such as *S. epidermidis*, *Klebsiella pneumoniae*, *S. aureus*, etc., and accelerated wound healing (Moydeen et al., 2019). Contardi et al. synthesized fibrous hydrogels based on PVP and hydroxycinnamic acid derivatives by electrospinning technique. *In vitro* experiments confirmed the release of antioxidants during 8 days, which protected A549 epithelial cells against oxidative stress. In addition, the biocompatibility of fibrous hydrogels was proved by using HaCaT, A549 cell lines, and red blood cells. Likewise, *ex vivo* studies showed that these hydrogels control the inflammatory phase and regenerate human skin (Contardi et al., 2021). According to these results, PVP can be used as a synthetic biomaterial with desirable properties in wound dressings.

## 4 Biomaterial-based wound dressing quality controls

The expansion of modern wound dressings based on biomaterials makes it necessary to determine how suitable they are for skin repair and regeneration. Hence, the quality control techniques play an important role in this way (Linares-Gonzalez et al., 2021). The produced wound dressings are first evaluated in the *ex vivo* environment and if their response is positive, they are measured for further investigation in the *in vivo* and preclinical environment. Currently, there are many techniques to determine and control the quality of dressings based on biomaterials *in vitro* and *in vivo*, such as functional analysis, histology through light and electron microscopes, biomechanical tests, and others. Carrying out all these studies is a prerequisite for obtaining approval from public-private organizations for the use of manufactured dressings (Zhang et al., 2020b; Sierra‐Sánchez et al., 2020; Boyce and Kagan, 2023).

*In vitro* assays on biomaterial-based dressings should confirm cell viability and their function. Besides, cell implantation on these dressings should restore the main functions of the skin histologically (Przekora, 2020). One of the first methods to evaluate cell viability was the use of trypan blue solution. Based on this method, cells are mixed with trypan blue solution and placed on neobar slide or hemocytometer for cell counting and determination of concentration. Dead cells are seen in blue color due to damage to their membrane, which differentiates them from living cells (Crowley et al., 2016; Kerschbaum et al., 2021). Over time, newer assays for cell viability were noticed. MTT assay, as the most known of these approaches, is used to measure the metabolic activity of cells. In this method, 3-(4,5-dimethylthiazol-2-yl)-2,5-diphenyltetrazolium bromide dye (called MTT) is used to evaluate cell viability. MTT is seen in yellow color in the neutral state. When placed on a dead cell, due to the presence of nicotinamide adenine dinucleotide phosphate (NADPH)-dependent cellular oxidoreductase enzyme, it turns into formazan, which emits a purple color. Therefore, it is possible to distinguish living cells from dead ones (Grela et al., 2018; Kumar et al., 2018; Mazloum-Ardakani et al., 2019).

*In vivo* assays are essential to prove the efficacy or failure of manufactured dressings. These quality control assays should indicate whether the produced dressings are biomimetic, bioadhesive, and biointegrated and whether they support epithelial tissue growth and maturation. Hence, studying these measurements to find answers to these questions is not without grace (Tithito et al., 2023). H&E staining is used as one of the common techniques to determine the effectiveness of skin dressings. This technique provides key information about biomaterials (biodegradation, rejection/acceptance, and encapsulation), granulation tissue thickness, angiogenesis, and ECM regeneration. In addition, the H&E technique allows researchers to identify many cells involved in wound healing such as fibroblasts, lymphocytes, granulocytes (basophils, eosinophils, and neutrophils), macrophages, and plasma cells (Miyazaki et al., 2019; Van De Vlekkert et al., 2020; Lu et al., 2021). Besides, other staining methods such as Masson’s trichrome, orcein, PAS, and picrosirius are used to identify the distinction between biomaterials and tissue. These techniques enable the confirmation of ECM reorganization and organization and the identification of abnormal synthesis, which is very effective for synthetic biomaterial-based dressings (Shefali et al., 2020; Ayyanar and Mitra, 2023).

## 5 Conclusion

Wounds are created on the skin in different forms (such as burns, tears, etc.). The natural wound healing process is a biological, dynamic, and time-consuming process that usually takes between 8 and 12 weeks. In the past, tree leaves, cloth, and sterile gauze were used to heal skin wounds. These substances not only did not reduce the healing time of the wound but also caused microbial and bacterial infection on the surface of the wound. With the advancement of science and technology in the field of medicine and skin tissue engineering, new wound dressings based on natural or synthetic biomaterials have been designed and produced with the aim of reducing wound healing time and preventing wound infection. Compared to traditional dressings, these dressings have many advantages, such as greater durability, hydrophilicity, low toxicity, biocompatibility, biodegradability, antibacterial properties, and antimicrobial effects. The existence of different sources, various synthetic methods, and increasing costs have brought many challenges to researchers using biomaterials. Although the extraction of most biomaterials from biomass has been made possible today, the economic and health debates of society have caused these biomaterials to be limited for medical and therapeutic uses. On the other hand, diverse sources around the world and traditional synthesis methods are long and expensive, making the use of biomaterials as wound dressings difficult. Focusing on natural biomaterials, this study introduced sources, synthesis methods, and application of biomaterials as wound dressings and discussed quality control techniques of produced dressings. In addition, the advances and limitations of biomaterials used in wound management were reviewed. However, it is suggested that future researchers consider how to solve the upcoming challenges and introduce a suitable solution for different wounds in their investigations. Today, most wound dressings are prepared and used through the electrospinning, pressurized gyration, etc. method. Since technology and medicine are developing and changing together, the use of new advanced technologies including microfluidics, dECM-based dressings, and 3D bioprinting can be interesting topics to investigate in the field of wound management.
